# NIR-II nanoprobes for investigating the glymphatic system function under anesthesia and stroke injury

**DOI:** 10.1186/s12951-024-02481-w

**Published:** 2024-04-23

**Authors:** Bin Sun, Danlan Fang, Wenzhong Li, Mengfei Li, Shoujun Zhu

**Affiliations:** 1 Joint Laboratory of Opto-Functional Theranostics in Medicine and Chemistry, First Hospital of Jilin University, Changchun, 130021 China; 2 State Key Laboratory of Supramolecular Structure and Materials, Center for Supramolecular Chemical Biology, College of Chemistry, Jilin University, Changchun, 130012 China

**Keywords:** Glymphatic system in rodent model, NIR-II fluorescence imaging, Glymphatic enhancer dexmedetomidine, Anesthesia, Stroke

## Abstract

The glymphatic system plays an important role in the transportation of cerebrospinal fluid (CSF) and the clearance of metabolite waste in brain. However, current imaging modalities for studying the glymphatic system are limited. Herein, we apply NIR-II nanoprobes with non-invasive and high-contrast advantages to comprehensively explore the function of glymphatic system in mice under anesthesia and cerebral ischemia–reperfusion injury conditions. Our results show that the supplement drug dexmedetomidine (Dex) enhances CSF influx in the brain, decreases its outflow to mandibular lymph nodes, and leads to significant differences in CSF accumulation pattern in the spine compared to isoflurane (ISO) alone, while both ISO and Dex do not affect the clearance of tracer-filled CSF into blood circulation. Notably, we confirm the compromised glymphatic function after cerebral ischemia–reperfusion injury, leading to impaired glymphatic influx and reduced glymphatic efflux. This technique has great potential to elucidate the underlying mechanisms between the glymphatic system and central nervous system diseases.

**Graphical Abstract:**

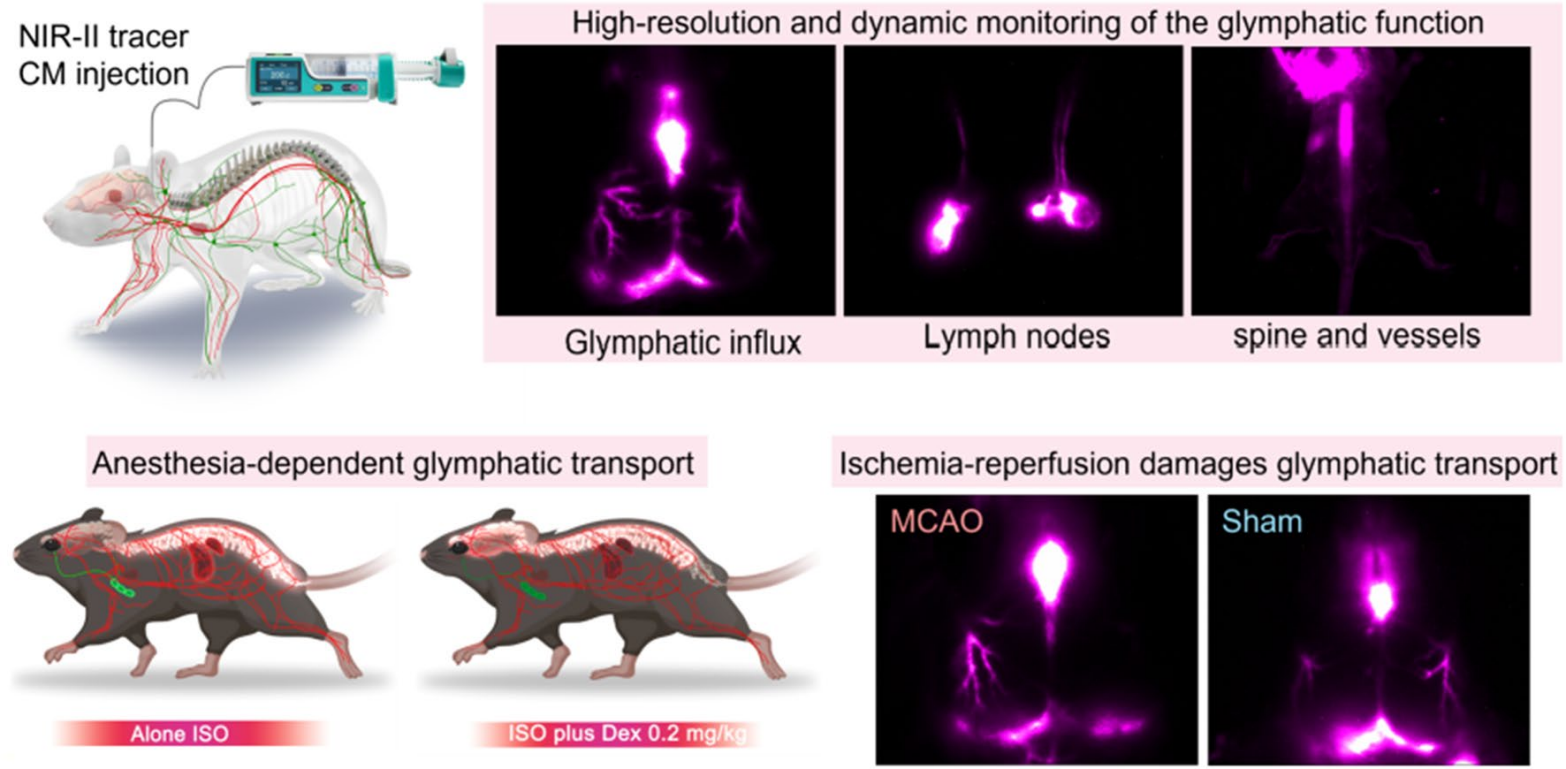

**Supplementary Information:**

The online version contains supplementary material available at 10.1186/s12951-024-02481-w.

## Introduction

The glymphatic system is a significant discovery in the field of brain sciences. It is considered as a pathway that allows the influx and egress of cerebrospinal fluid (CSF) in the brain, responsible for clearing harmful proteins and metabolites generated in the brain parenchyma through CSF circulation [1–6]. The perivascular spaces (PVS) surrounding the vessel and lined by astrocytic endfeet are the main component of the glymphatic system. This system is categorized into periarterial spaces and perivenous spaces. Periarterial spaces facilitate the inflow of CSF into the brain, while perivenous spaces provide channels for CSF outflow from the brain. Hence, the PVS is one of major factors for the determination of the glymphatic system function. The astrocytic endfeet construct a semi-permeable barrier that enables the CSF transport between PVS and brain parenchyma, thus also influencing the efficacy of glymphatic system’s function. It has been established that the glymphatic system is involved in central nervous system (CNS)-related diseases such as Alzheimer’s disease, traumatic brain injury, stroke, and Parkinson's disease [6–9]. Impairment of the glymphatic system can lead to inefficient clearance of harmful proteins and metabolites, resulting in their accumulation in the brain parenchyma and worsening of CNS-related diseases [10–14]. Especially, glymphatic system serves as a therapeutic target for the treatment of CNS-related diseases, such as traumatic brain injury [6, 15]. Therefore, it is crucial to understand the known and unknow factors influencing the glymphatic system function. This includes understanding how glymphatic function varies under different physiological and pathological conditions, which is vital for advancing treatments for CNS-related diseases. Anesthesia has been reported to modify glymphatic transport through affecting the brain’s state, for instance, isoflurane (ISO) anesthesia suppresses glymphatic influx, while additional administration of dexmedetomidine (Dex) has been shown to augment glymphatic influx by decreasing norepinephrine levels and increasing delta band power [16, 17]. However, the overarching impact of Dex on glymphatic clearance and systemic CSF circulation has yet to be fully elucidated. Meanwhile, the exact functional changes in glymphatic influx and glymphatic clearance following ischemia–reperfusion injury remain unclear. It is particularly important to define the change in glymphatic clearance post-ischemia–reperfusion injury, as impaired glymphatic clearance could reduce the efflux of metabolic wastes from brain parenchyma, leading to the accumulation of proteins and toxic substances.

Studies on exploring the anatomical structure and evaluating the function of the glymphatic system have been conducted in both clinical and basic research using various imaging modalities. These include magnetic resonance imaging (MRI) [18], brain section microscopy [19], two-photon microscopy [3], and transcranial microscope imaging in basic research [20–22]. Studies based on these imaging modalities have contributed to a better understanding of the interaction between the glymphatic system and central nervous system disorders. However, they still face some challenges, including (i) The large invasiveness caused by the preparation of brain sections. (ii) The use of general microscope imaging tools for studying the glymphatic system function suffers from low resolution, low signal-to-background ratio (SBR), and invasiveness. (iii) These imaging techniques are incapable of real-time imaging the body-wide CSF tracer circulation [6, 23, 24]. Therefore, the development of an imaging modality that achieves higher clarity, lower invasiveness and body-wide scale monitoring of CSF dynamic is important and useful for investigating glymphatic system function in rodent animal models.

**Scheme 1:**
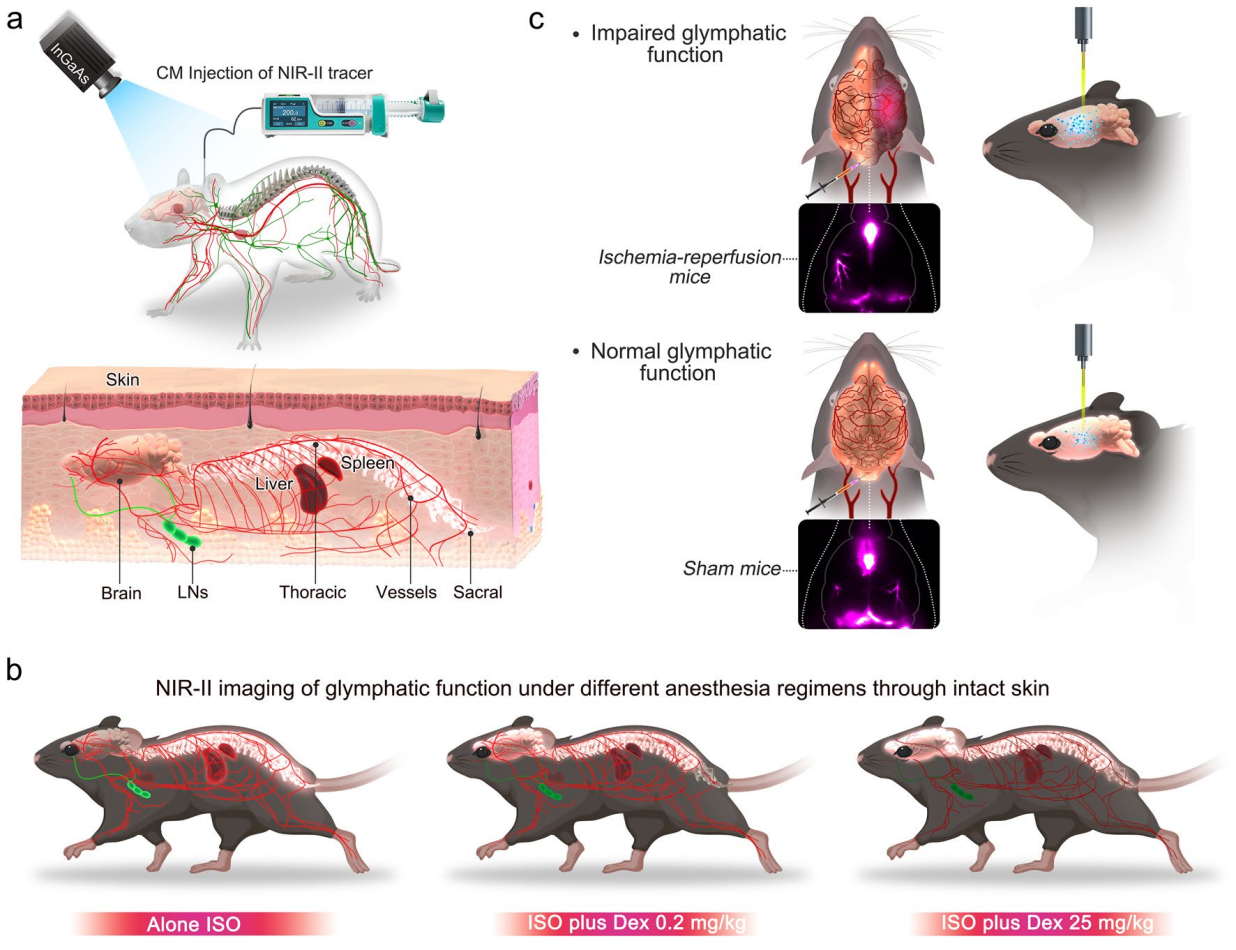
NIR-II fluorescence imaging enables in vivo imaging of the function of the glymphatic system under different physiological and pathological conditions. **a** Schematic of the NIR-II tracer infusion into cisterna magna (CM) and noninvasive imaging of CSF circulation pathway through intact skin. **b** High-resolution detection of the patterns of anesthesia-dependent CSF distribution. **c** NIR-II imaging visualizes the impaired glymphatic influx and clearance function after ischemia–reperfusion injury. The schematic diagram was created by using the online software https://biorender.com

Herein, we utilized the NIR-II nanoprobes [25] (BSA@IR-780 and Quantum dots) to dynamically visualize the modulation of CSF influx in the brain and the patterns of CSF clearance through mandibular lymph nodes (mLNs) and the spine into the blood circulation system under different anesthesia regimens (Scheme 1a, b). Subsequently, we investigated the contentious yet important question of the glymphatic-stroke interaction (Scheme 1a, c). The high-penetration NIR-II fluorescence signal allowed us to rapidly evaluate the size of the endfoot tube and the influx of the NIR-II tracer into the brain [26, 27]. Our findings demonstrate that the use of dexmedetomidine (Dex) as a supplement drug for inhalational isoflurane (ISO) anesthesia enhances CSF influx in the brain. The clearance of the cisterna magna (CM)-injection tracer into the blood circulation system is greater under ISO anesthesia alone than under ISO anesthesia supplemented with Dex. Our experiments highlight the potential of NIR-II imaging as a promising modality for studying the glymphatic system in rodent animal models and evaluating its potential as a target for the diagnosis and treatment of stroke.

## Results and discussion

### NIR-II fluorescence imaging enabled high-contrast imaging of glymphatic system in a rodent model

**Fig. 1:**
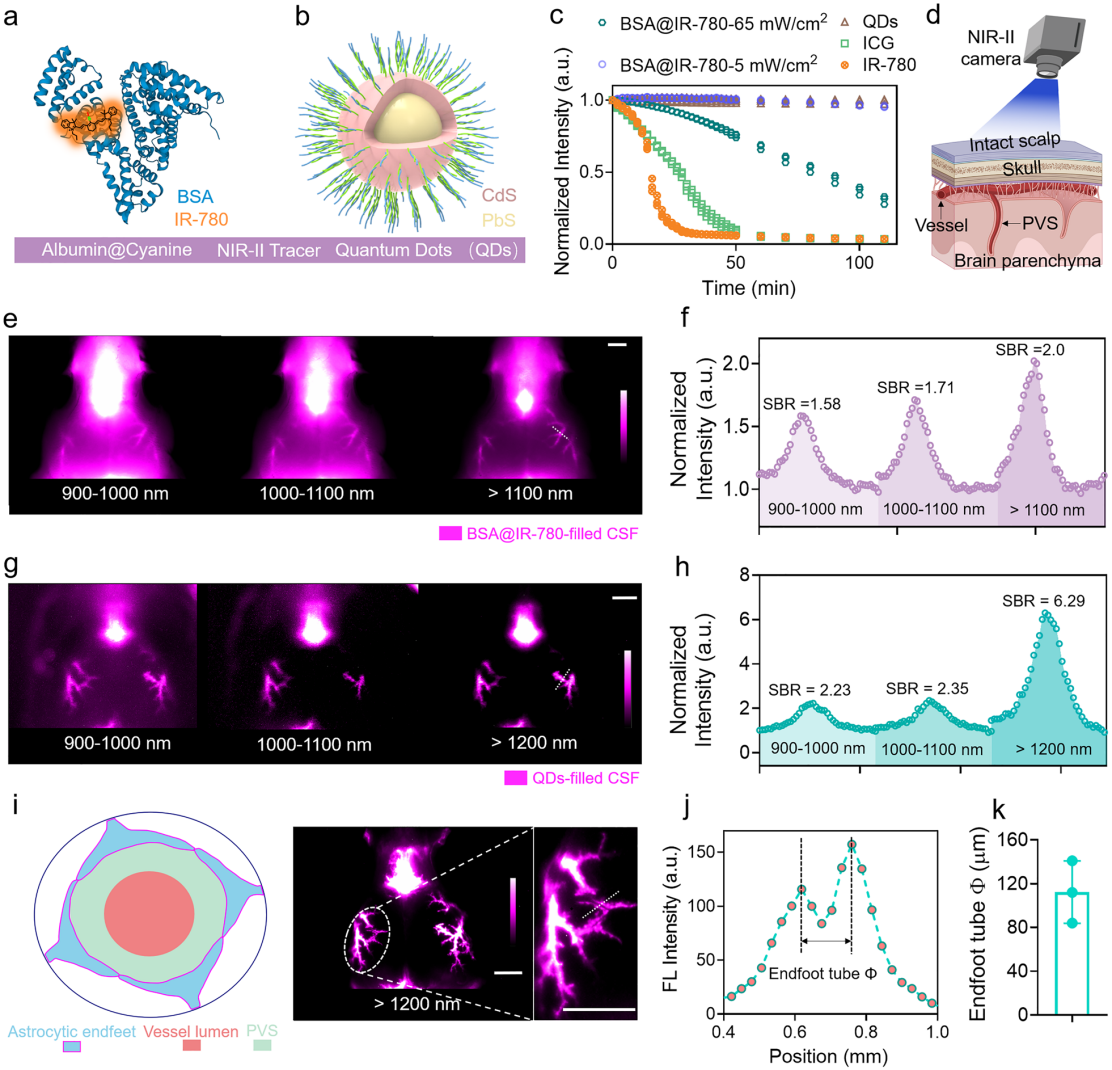
Utilizing NIR-II nanoprobes for high-contrast imaging of the CSF flow along PVS. **a-b** Schematic representation of selective NIR-II fluorophores (BSA@IR-780 and QDs) as CSF tracers. Blue represents BSA protein. Orange represents IR-780 dye. Rose red represents CdS shell. Yellow represents PbS core.** c** The NIR-II tracers BSA@IR-780 and QDs demonstrated superior photostability compared to cyanine dyes. **d** The cartoon depicts the collection of images through the intact scalp. The schematic diagram was created by using the online software https://biorender.com. **e–h** Both BSA@IR-780 and QDs allowed for high-contrast imaging of CSF flow along the MCA through intact scalp. White scale bar: 2 mm. Fluorescence signal along the white dashed line inserted in e and g was collected. Fluorescence signal was normalized with the smallest signal value along the white dashed line. **i** The NIR-II tracer QDs enabled visualization of the size of the endfoot tube. White scale bar: 2 mm. Blue represents astrocytic endfeet. Pink represents vessel lumen. Green represents PVS. **j** Cross-sectional fluorescence signal along the white dashed line inserted in **i** was collected to quantify the size of the endfoot tube **k**.The colored dots indicating three adjacent regions were measured by using profiles tool in Fiji software

We selected two types of NIR-II nanoprobes, BSA@IR-780 and quantum dots (QDs), as CSF tracers and evaluated their ability to image the glymphatic system (Fig. 1a, b and Additional file 1: Figs. S1–S3) [28–33]. The BSA@IR-780 was chosen because it can mimic macromolecules in CSF, while QDs were selected for their superior imaging quality and comparable circulation patterns as BSA@IR-780. We compared the photostability of BSA@IR-780 and QDs with the clinically-used ICG and found that both BSA@IR-780 and QDs exhibited sufficient stability under continuous irradiation (Fig. 1c). By using a laser intensity of 5 mW/cm^2^, we achieved even better photostability for BSA@IR-780 tracer compared with the photostability under a laser intensity of 65 mW/cm^2^. The intensity of 5 mW/cm^2^ is the power density used for imaging the brain slices in the following sections.

Next, we delivered BSA@IR-780 and QDs into the cisterna magna (CM) using a micro-injection pump to measure the image contrast through the intact scalp (Fig. 1d) in rodent models. Moreover, we used young male C57BL/6 mice weighing between 21 and 25 g at postnatal 6–8 weeks to conduct all experiments. Under 808 nm excitation, we used filters to collection the fluorescence signal within different wavelengths (900 nm–1000 nm, 1000 nm–1100 nm, > 1100 nm) and the images showed that the MCA filled with BSA@IR-780 tracer was visualized in all imaging windows. The SBR value was used for assessment of imaging contrast and the SBR values were calculated by the highest fluorescence intensity divided the lowest fluorescence intensity along the white dash (Fig. 1e–h). Multiple wavelength bands were used for collecting images, aiming to test the imaging ability of selected NIR-II probes and to screen out the optimal imaging wavelength with both sufficient fluorescence signal and excellent imaging quality. Although the imaging contrast of BSA@IR-780 was lower than that of QDs, it was still reasonable for in vivo imaging and particularly important for ex vivo quantification using a commercial scanner (Fig. 1e–h). We measured the in vivo imaging quality of QDs at different wavelengths (900 nm–1000 nm, 1000 nm–1100 nm, > 1200 nm) through the intact scalp (Fig. 1g, h). The quantified results showed that the optimal imaging resolution was achieved over 1200 nm with the highest contrast (SBR = 6.29). After removing the scalp, the QDs exhibited even higher contrast (Additional file 1: Fig. S3b, c; SBR = 9.16 over 1200 nm). Interestingly, the removal of the scalp allowed for straightforward visualization of the PVS, and the high resolution enabled the measurement of the size of the endfoot tube located at the cortical surface (Fig. 1i, j) [34]. We measured the size of the endfoot tube using NIR-II images, which outperformed the frequently used two-photon microscopy with a complicated process and fluorescence microscopy with lower image resolution (Fig. 1k). The imaging quality was also tested by intralipid penetration experiment (Additional file 1: Fig. S4) [35, 36].

### In vivo* NIR-II imaging evaluated glymphatic system function and identified the Dex as an enhancer for glymphatic system function*

**Fig. 2:**
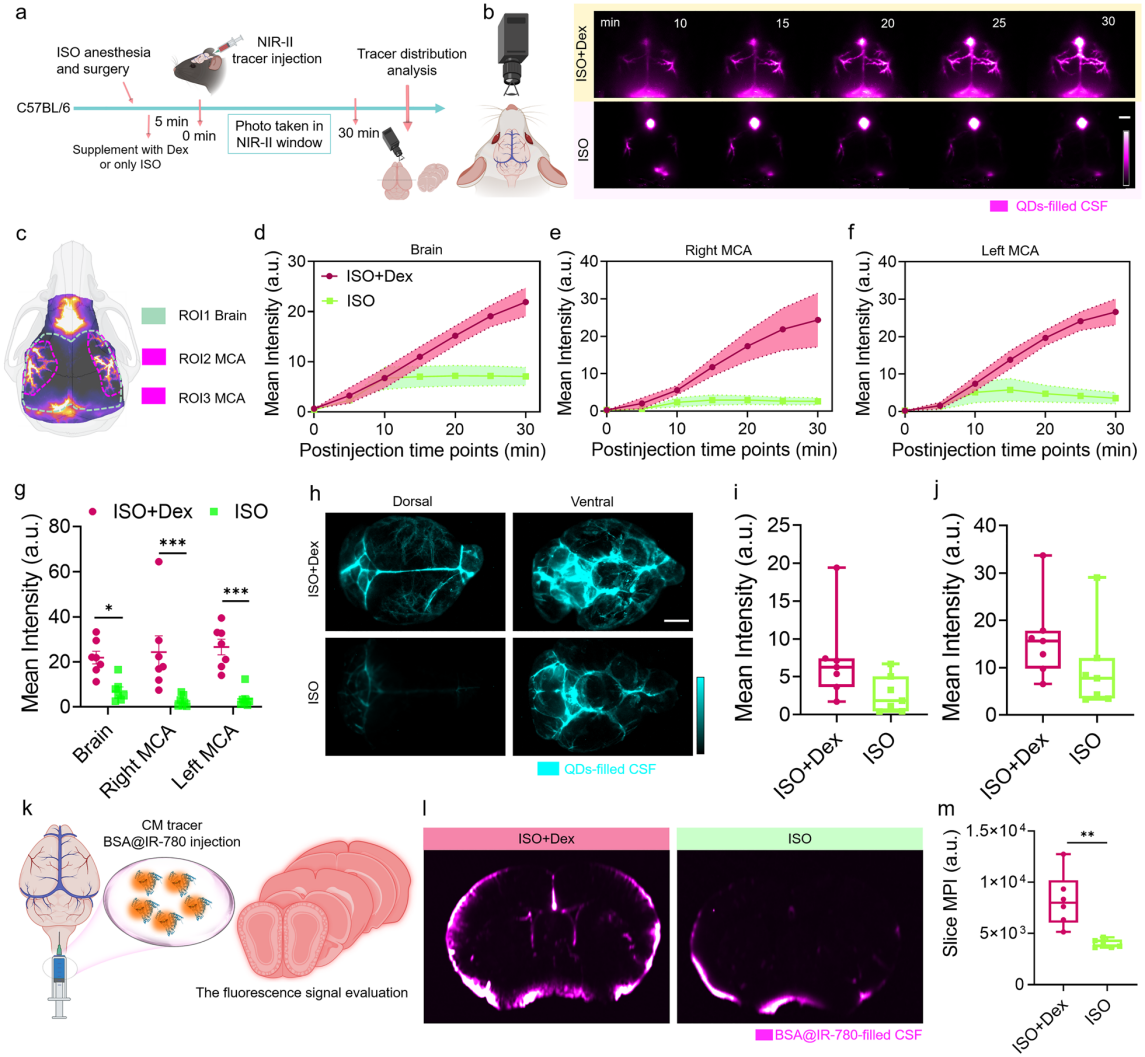
Noninvasive NIR-II fluorescence imaging of CSF distribution in the brain under ISO or ISO supplemented with Dex anesthesia. **a** Schematic representation of the NIR-II fluorescence imaging to investigate the effect of different anesthesia regimens on CSF influx in the brain. Dex dose is 0.2 mg/kg. The schematic diagram was created by using the online software https://biorender.com. **b** Brain imaging to record CSF distribution within 30 min after CM injection initiation. White scale bar: 2 mm. The schematic diagram was created by using the online software https://biorender.com. **c** The schematic shows the outline of three regions of interest (ROIs) at the dorsal brain position to quantify CSF distribution. The schematic diagram was created by using the online software https://biorender.com. The mean intensity variation of **d** Brain (ROI1), **e** right MCA (ROI2), and **f** left MCA (ROI3) over 30 min after CM injection initiation. Solid lines: means, shading: SEM. n = 7 mice per group. The region of mean intensity quantification was manually outlined according to the cartoon pattern in **c**. The mean intensity of MCA region represents the influx of NIR-II tracer along PVS surrounding the MCA. **g** Comparison of mean intensity at the 30 min time point. Two-Way ANOVA with Sidak’s multiple comparisons test, Brain: *P* = 0.0187, Right MCA: *P* = 0.0004, Left MCA: *P* = 0.0002, n = 7 mice per group. **h** Brain tissue imaging shows the distribution of the NIR-II tracer at the dorsal and ventral brain surfaces. Comparison of mean intensity in the two anesthetic mice at the dorsal **i** and ventral **j** positions, n = 7 mice per group, *t* test, Dorsal:* P* = 0.0733, Ventral:* P* = 0.2081. White scale bar: 2 mm. The quantified region was shown in Fig. S7a. Colored dot represents the intensity of individual animal. Center is median and quartiles shown by box and whiskers.** k** Schematic illustration represents the CM injection of BSA@IR-780 and analysis of coronal slice distribution. The schematic diagram was created by using the online software https://biorender.com.** l** The representative images reveal the distribution of the BSA@IR-780 within slices.** m** Comparison of mean slice intensity across all anesthetic mice, n = 6 mice per group, *t* test, *P* = 0.029. Colored dot represents the intensity of individual animal. Center is median and quartiles is shown by box and whiskers

To determine whether this imaging technology has the potential to achieve real-time and dynamic assessment of the glymphatic system function, we monitored the well-established effect of anesthetic drugs on the glymphatic influx. ISO anesthesia inhibits the CSF influx in the brain, while Dex is used as a supplementary drug and is transported by intraperitoneal injection at a certain dose to enhance the glymphatic influx in the brain [16, 17, 37–39]. We primarily used NIR-II imaging to evaluate the enhancing role of Dex at a twice-daily administration dose of 0.015 mg/kg based on a previous report and we also tested the effect of different doses of Dex on the glymphatic system [17]. Figure 2a illustrates the experimental process for evaluating the effect of different anesthesia regimens on CSF influx in the brain. ISO anesthesia was maintained throughout the experiment, including surgery, NIR-II tracer injection, and NIR-II imaging. For the ISO plus Dex groups, selective doses of Dex (25 mg/kg, 0.015 mg/kg*2, and 0.2 mg/kg) were intraperitoneally administered five minutes before the start of CM injection. For the 0.015 mg/kg*2 group, the mice received twice injection at 30 and 5 min respectively before the start of infusion. The CSF circulation pathway was then monitored by detecting the NIR-II signals through the intact scalp [17, 38].

The movement of CSF along the PVS surrounding the middle cerebral artery (MCA) has been considered as an imaging marker for evaluating variations in glymphatic system function in the brain-related diseases and circadian rhythms [4, 40–42]. Figure 2b and Additional file 1: Fig. S6 show representative time-course images of NIR-II tracer distribution at the dorsal surface of the brain under different anesthesia regimens. The PVS surrounding the MCA exhibited significant differences in brightness, with the ISO anesthesia group showing low brightness and the ISO plus Dex (different doses) anesthesia groups showing higher brightness (Fig. 2b and Additional file 1: Fig. S6a). The dorsal surface of the brain was artificially divided into three regions of interest (brain region, right MCA region, and left MCA region) to quantify the change in NIR-II signal across all time points (Fig. 2c). The quantified results further confirmed the inhibited glymphatic influx in the brain under ISO anesthesia and the improved glymphatic influx in the brain with additional Dex supplementation (Fig. 2d–f and Additional file 1: Fig. S6b-d).

Afterwards, we collected the brain tissues and assessed the distribution of CSF tracer QDs on the dorsal and ventral brain surfaces. The images revealed a significant increase in NIR-II signals in the ISO plus Dex groups compared to the ISO group (Fig. 2h–j and Additional file 1: Fig. S7). However, there were no significant signal differences between different doses of Dex (Additional file 1: Fig. S8). To thoroughly study the NIR-II tracer influx in the brain under different doses of Dex, we utilized BSA@IR-780 to evaluate the NIR-II tracer distribution within brain slices under selective anesthesia regimens (Fig. 2a–k). The brains were harvested after 30 min of circulation, and coronal slices were prepared according to the designated sites shown in the diagram (Additional file 1: Fig. S9a). The mean intensity across twelve slices yielded results consistent with the in vivo imaging, indicating that supplementing ISO with Dex enhances CSF tracer influx in the brain surface and brain parenchyma (Fig. 2l, m, and Additional file 1: Fig. S9).

### Administration of Dex modulated the clearance of CSF

**Fig. 3:**
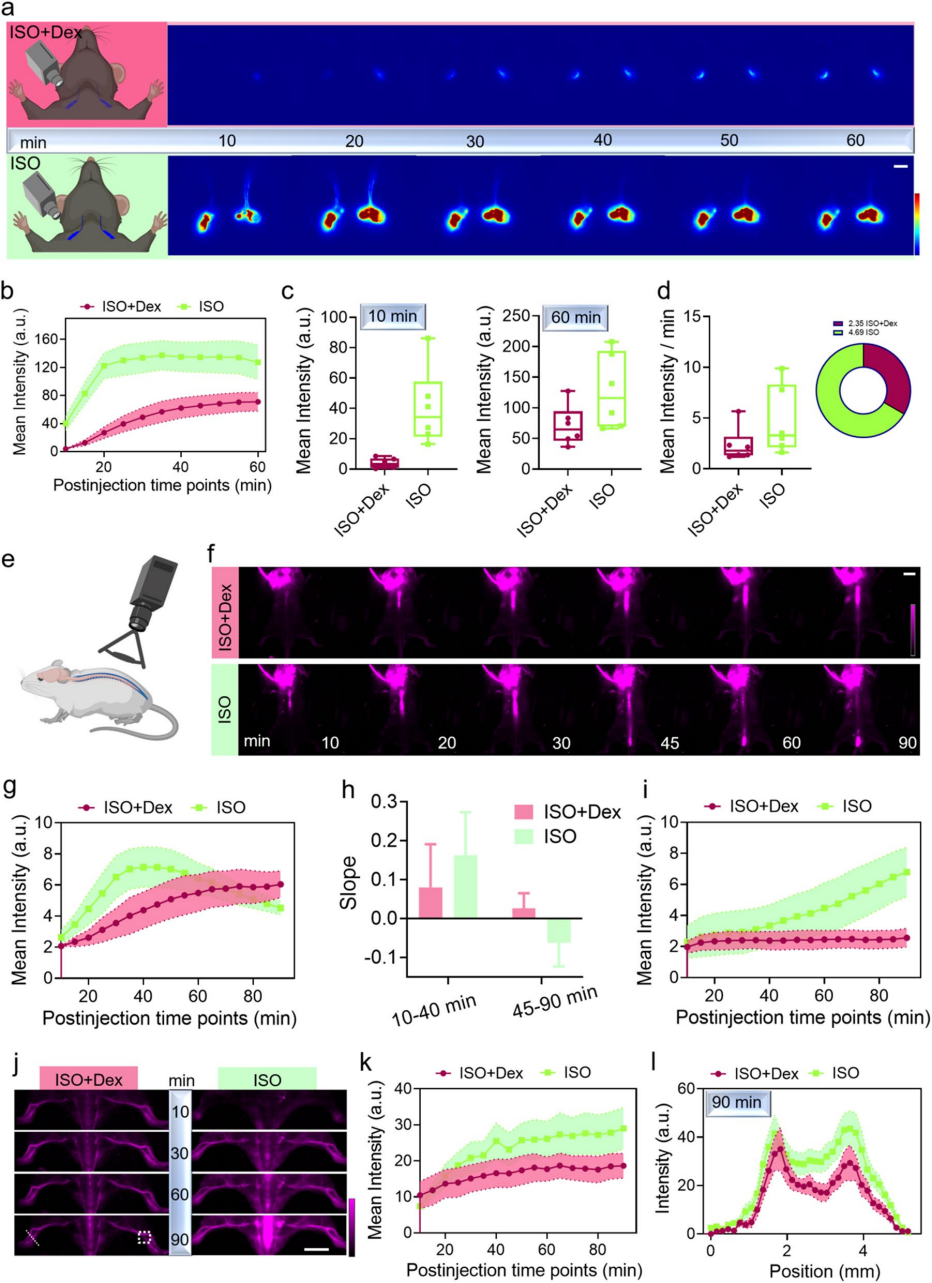
NIR-II fluorescence imaging of the effect of anesthesia on CSF clearance. **a** Schematic of local LNs imaging in the NIR-II window through intact neck skin and time-course imaging of LNs over 60 min to record the signal variation of NIR-II tracer-filled LNs under ISO anesthesia or ISO supplemented with Dex. Dex dose is 0.2 mg/kg. White scale bar: 2 mm. The schematic diagram was created by using the online software https://biorender.com. **b** Mean fluorescence intensity over 60 min after the start of CM injection.** c** Comparison of LNs intensity at 10 min and 60 min after CM injection initiation, *t* test, 10 min:* P* = 0.0057, 60 min:* P* = 0.0731. Colored dot represents the intensity of individual animal. Center is median and quartiles shown by box and whiskers. **d** Comparison of LN filling rate with NIR-II tracer calculated from 10 to 30 min after CM injection initiation between two anesthesia regimens, n = 6 mice per group, *t* test, *P* = 0.1568. Colored dot represents the filling rate of individual animal. Center is median and quartiles shown by box and whiskers. **e** Schematic of spine imaging in the NIR-II window through intact skin. Dex dose is 0.2 mg/kg. The schematic diagram was created by using the online software https://biorender.com.** f** Representative images of spine imaging over 90 min to monitor CSF distribution. White scale bar: 1 cm. **g** Mean fluorescence intensity variation in the thoracic region over 90 min, n = 8 for ISO plus Dex group, n = 6 for ISO group. **h** Comparison of QDs-filling rate in the thoracic region before 40 min and after 45 min, n = 8 for ISO plus Dex group, n = 6 for ISO group. **i** Mean fluorescence intensity variation in the sacral region, n = 6 for ISO plus Dex group, n = 6 for ISO group. **j** Representative images showing increasing fluorescence signal in hindlimb vessels after CM injection. The ISO group exhibited a faster intensity increase and brighter hindlimb vessel outlines compared to the group supplemented with Dex. White scale bar: 1 cm. **k** Quantification of fluorescence signal variation in hindlimb vessels, n = 8 for ISO plus Dex group, n = 6 for ISO group. **l** Comparison of hindlimb vessel intensity at the 90 min time-point, n = 8 for ISO plus Dex group, n = 6 for ISO group

After allowing the tracer circulation with CSF, we clearly visualized the peripheral lymphatic system through intact skin. The cervical (mandibular) and sacral lymph nodes (LNs), which are known as important clearance pathways for CSF, were both illuminated, and the lymphatic vascular networks connected to these LNs were also mapped with high SBR (Additional file 1: Figs. S10, S11) [40, 43, 44]. Quantitative analysis showed that ISO anesthesia alone demonstrated the highest fluorescence signal, and the fluorescence signals were inhibited when supplemented with Dex (Additional file 1: Fig. S10a, c). Next, we conducted a detailed study on the correlation between different doses of Dex and lymphatic drainage by performing time-course NIR-II images of the mandibular LNs. We found that the LNs of mice under ISO anesthesia alone emitted the highest fluorescence signals at all time points (Fig. 3a, b and Additional file 1: Fig. S12), especially at the first time point when the LNs were filled with numerous CSF tracers (Fig. 3c and Additional file 1: Fig. S13a). This suggested that ISO anesthesia lead to an enhancement of the flow of NIR-II tracer into the LNs after CM injection.

Because we have confirmed the Dex facilitating glymphatic influx, we speculated that Dex might reduce the CSF outflow into the peripheral lymphatic system. As expected, the introduction of Dex to supplement ISO anesthesia significantly reduced the fluorescence signal from the LNs (Fig. 3a, c, Additional file 1: Figs. S12, S13). Therefore, we measured the effect of different doses of Dex on lymphatic drainage and compared the rate of LNs filling with NIR-II tracer during the first 20 min of recording. The results showed that under a dose of 25 mg/kg, the LNs had the lowest fluorescence signal and filling rate (Fig. 3a–d and Additional file 1: Figs. S12, S13). Combining the results of slice images and in vivo brain imaging, we concluded that administering Dex at a dose of 25 mg/kg to support ISO anesthesia had a significant effect on enhancing CSF influx in the brain and slowing the movement of CSF into the mandibular LNs compared to doses of 0.015 mg/kg*2 and 0.2 mg/kg. However, we considered 0.2 mg/kg to be a suitable dose to support ISO anesthesia because the significant reduction of lymphatic drainage could increase the risk of metabolic waste accumulation in the brain parenchyma.

The drainage of mandibular LNs does not represent the entire glymphatic efflux because the spine and spine-related LNs are important pathways for the clearance of CSF [43, 45]. To investigate this, we performed in vivo monitoring of the distribution of NIR-II tracer in the spine, as well as its travel into the blood circulation (Fig. 3e). Under ISO anesthesia alone, the NIR-II tracer initially accumulated in the thoracic region and gradually accumulated in the sacral region. However, under ISO anesthesia supplemented with Dex (0.2 mg/kg), the NIR-II tracer mainly accumulated in the thoracic region throughout the 90 min (Fig. 3f). From the intensity curve of the sacral region, we confirmed that the CM-injected NIR-II tracer could travel from the thoracic region to the sacral region within 90 min under ISO anesthesia, while it hardly accumulated in the sacral region under ISO anesthesia supplemented with Dex (0.2 mg/kg) (Fig. 3g–i). For the 25 mg/kg Dex dose, the curve also displayed a pattern of first rising and then falling, similar to the ISO alone group in the thoracic region. However, there was a lower filling rate and less accumulation of the NIR-II tracer in the thoracic and sacral regions compared to the ISO alone groups (Additional file 1: Fig. S14).

Because both the mandibular lymph nodes and spine are major clearance pathways for CSF and exhibit distinct CSF accumulation under selective anesthesia regimens, we measured the dynamic clearance of NIR-II tracer after CM injection initiation by monitoring the intensity variation in hindlimb vessels and whole-body vessels. We first compared the effect of ISO alone and ISO supplemented with Dex (0.2 mg/kg) groups (Fig. 3j), and the fluorescence signal continuously increased for both groups after CM injection initiation. The plotted fluorescence intensity curve representing the vascular network indicated that clearance was higher in the ISO group compared to the groups supplemented with Dex (Fig. 3k, l). Since the QDs in the blood system are mainly taken up by the liver and spleen, the quantified intensity of the liver and spleen in the ISO group had the highest fluorescence intensity, while the groups supplemented with Dex 25 mg/kg had the lowest fluorescence intensity (Additional file 1: Fig. S15). All the results suggested that ISO anesthesia reduced the influx of NIR-II tracer in the brain, but it did not strongly reduce the clearance of NIR-II tracer delivered by CM injection. Overall, the suitable Dex dose for mice weighing 20–25 g in our experiment was 0.2 mg/kg, as this dose enhanced glymphatic influx without strongly reducing CSF clearance.

### NIR-II fluorescence imaging revealed impaired glymphatic function after ischemia–reperfusion injury

**Fig. 4:**
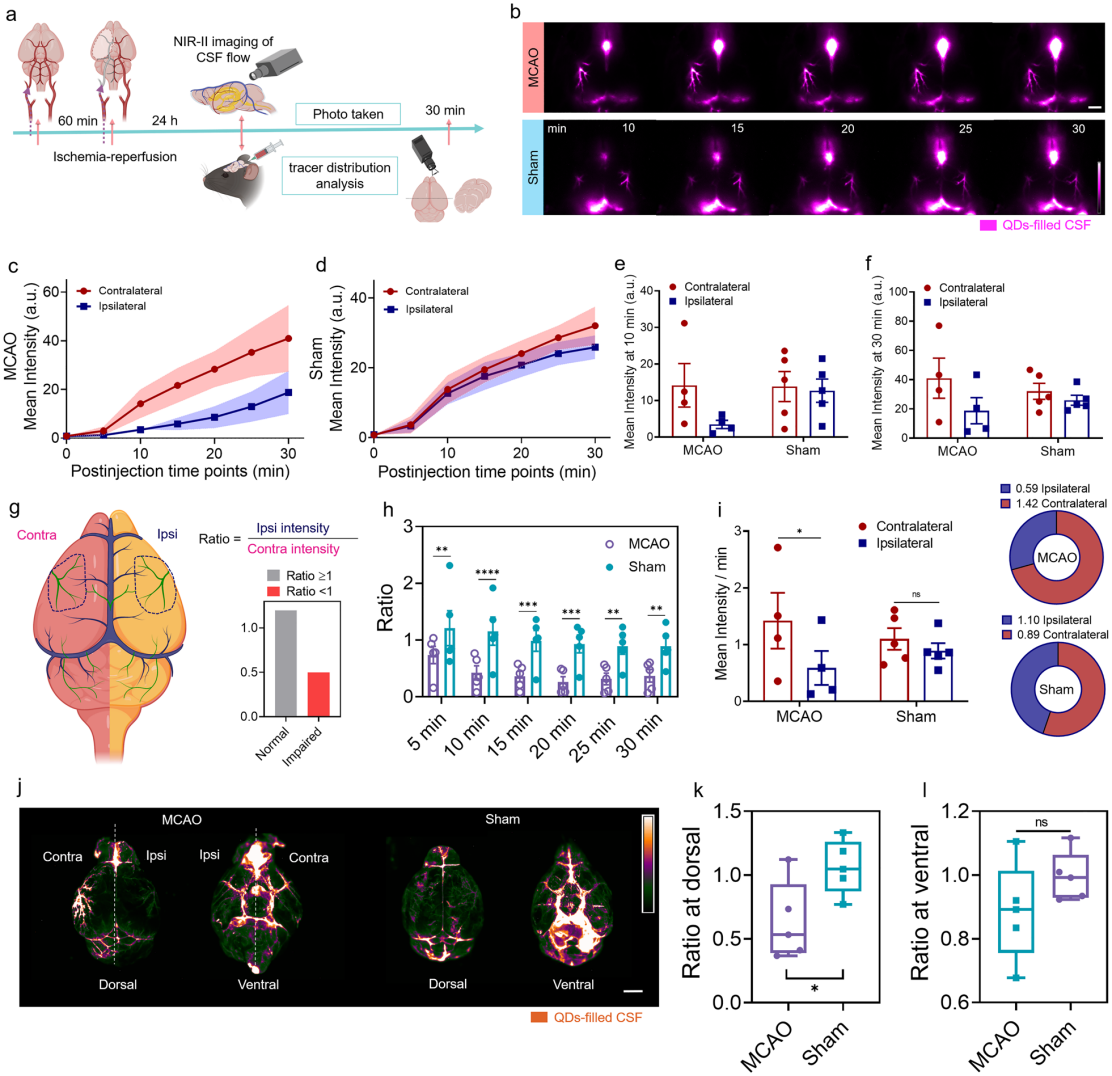
Evaluation of glymphatic system function in the ischemia–reperfusion stroke model using NIR-II fluorescence imaging. **a** Workflow of the in vivo imaging experiment. The schematic diagram was created by using the online software https://biorender.com. **b** Representative NIR-II images showing impaired glymphatic system function after MCAO and undamaged glymphatic function in sham mice. Time-series of fluorescence signal variation in each hemisphere of MCAO mice **c** and sham mice **d**. White scale bar: 2 mm. Comparison of mean intensity between contralateral hemisphere and ipsilateral hemisphere at 10 min **e**, two-way ANOVA with Sidak’s multiple comparisons test, MCAO: *P* = 0.1758 < Sham: *P* = 0.9727 and 30 min **f**, two-way ANOVA with Sidak’s multiple comparisons test, MCAO: *P* = 0.1658 < Sham: *P* = 0.8214, n = 4 for MCAO group, n = 5 for sham group. **g** Schematic illustrating the quantification process of the ratio. The schematic diagram was created by using the online software https://biorender.com. **h** Time-series of ratio within 30 min after CM infusion initiation in MCAO mice and sham mice, n = 5 per group, two-way ANOVA with Sidak’s multiple comparisons test, 5 min: *P* = 0.0085, 10 min: P < 0.0001, 15 min: *P* = 0.0005, 20 min: *P* = 0.0002, 25 min: *P* = 0.0012, 30 min: *P* = 0.0037. **i** The NIR-II tracer filling rate in MCA region in MCAO mice and sham mice calculated from the intensity variation within 30 min duration, n = 4 for MCAO group, n = 5 for sham group, two-way ANOVA with Sidak’s multiple comparisons test, MCAO: *P* = 0.0219, Sham: *P* = 0.5736. **j** Imaging of the NIR-II tracer distribution on the brain surface at the dorsal and ventral positions. White scale bar: 2 mm. The ratio of MCAO mice and sham mice at the dorsal **k** and ventral **l** positions, n = 5 per group, *t* test, Dorsal: *P* = 0.0336, Ventral: *P* = 0.1951, Colored dot represents the ratio of individual animal. Center is median and quartiles shown by box and whiskers

We investigated whether reperfusion injury affects the glymphatic system (Fig. 4a). TTC staining and H&E staining confirmed the successful preparation of the ischemia-reperfusion stroke mouse model (Additional file 1: Fig. S16a, b) [46]. After allowing ischemia–reperfusion for 24 h, time-course NIR-II images revealed a significant difference in CSF flow between the sham and MCAO groups (Fig. 4b). The NIR-II tracer moved more along the PVS pathway in the contralateral hemisphere than in the ipsilateral hemisphere in MCAO mice, and the mean fluorescence intensity in the contralateral hemisphere was approximately twice that in the ipsilateral hemisphere (Fig. 4c–f). Furthermore, the similar fluorescence signal variation between the contralateral and ipsilateral hemispheres in sham mice suggested that unimpaired function of the glymphatic system (Fig. 4c–f).

We proposed a ratio calculation method to evaluate the level of glymphatic system impairment. The ratio was calculated by dividing the ipsilateral MCA intensity by the contralateral intensity (Fig. 4g). A ratio greater than or equal to 1 indicates unimpaired glymphatic function, while a ratio less than 1 indicates glymphatic function impairment due to ischemia–reperfusion (Fig. 4h). The sham group exhibited a mean ratio close to 1 at all time points, while the MCAO group showed a significantly lower mean ratio, even at the initial 5 min time point (Fig. 4h). The eventual ratio of the sham group at the 30 min time point was approximately 2 times higher than that of the MCAO group (Fig. 4h). We also confirmed that the ischemia–reperfusion injury could significantly reduce the rate of PVS filling in the ipsilateral hemisphere compared to sham groups (Fig. 4i). Furthermore, we generated NIR-II images of brain tissues to confirm the higher ratio in sham mice compared to MCAO mice, both quantified from the dorsal and ventral brain surfaces (Fig. 4j–l). The inconspicuous ratio distinction between the sham and MCAO groups on the ventral brain surface may be due to the initial filling of the NIR-II CSF tracer in the Willis in the ventral brain after CM infusion, leading to a mass of tracer accumulation.

**Fig. 5:**
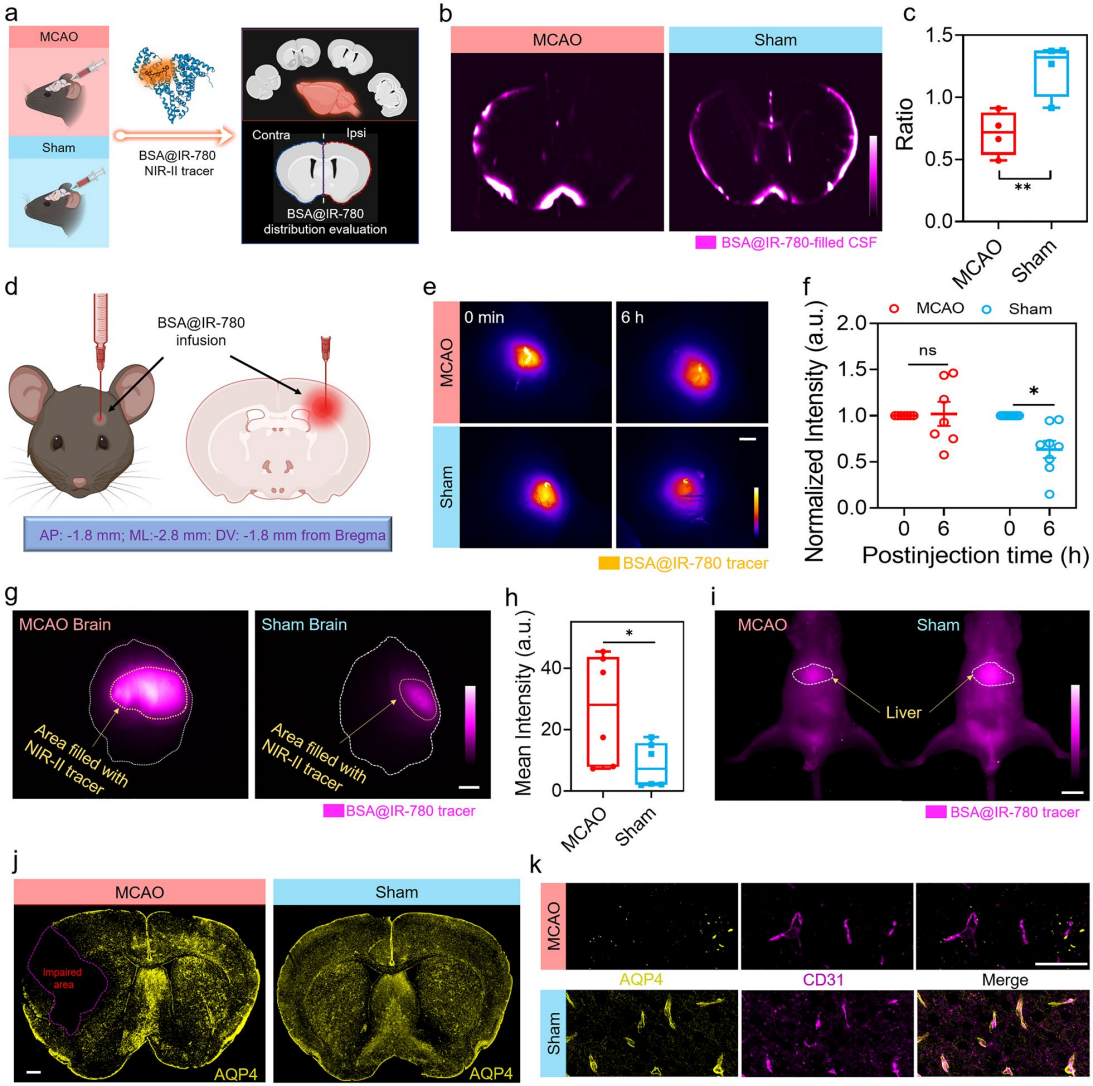
Impaired NIR-II tracer influx in the brain and clearance from the brain. **a** Schematic illustrating the evaluation of NIR-II tracer distribution using coronal brain sections. The schematic diagram was created by using the online software https://biorender.com. **b** Representative brain slice images showing uniform tracer distribution in sham mice and uneven distribution in MCAO mice. **c** Ratio of brain slices, n = 4 per group, *t* test, *P* = 0.0098. Colored dot represents the ratio of individual animal. Center is median and quartiles shown by box and whiskers. **d** Schematic depicting the experiment for evaluating brain clearance levels. The schematic diagram was created by using the online software https://biorender.com. **e** NIR-II imaging through the intact skull for comparing the remaining NIR-II tracer between the MCAO group and the sham group 6 h post-infusion. White scale bar: 2 mm. **f** Impaired brain clearance in MCAO mice compared to sham mice, n = 7 for MCAO group, n = 8 for sham group, two-way ANOVA with Sidak’s multiple comparisons test, MCAO: *P* = 0.9822, Sham: *P* = 0.0046. Fluorescence signal at 6 h time point was normalized with the fluorescence signal at 0 min time point. **g** Brain tissue imaging for evaluating the residual NIR-II tracer of MCAO mice and Sham mice. White scale bar: 2 mm. The whole brain was manually outlined by Fiji software to measure the mean intensity. **h** Comparison of mean intensity of brain tissues between MCAO mice and sham mice 6 h post-infusion. The mean intensity quantifying the residual NIR-II tracer, n = 6 per group, *t* test, *P* = 0.0425. Colored dot represents the intensity of individual animal. Center is median and quartiles shown by box and whiskers. **i** NIR-II imaging of liver after 6 h clearance, n = 3 per group. White scale bar: 1 cm. The liver was manually outlined by Fiji software to measure the mean intensity. **j-k** Immunohistochemistry analysis of MCAO-induced glymphatic function impairment. Yellow represents AQP4, magenta represents vessels, and the overlap between AQP4 and vascular staining. White scale bar: 500 µm for **j**, White scale bar: 50 µm for **k**

To further evaluate the effect on tracer distribution in coronal brain sections, we used BSA@IR-780 for CM injection in both the sham and MCAO groups (Fig. 5a). The effect on tracer distribution in coronal brain sections was quantified by calculating the ratio of mean fluorescence intensity in the ipsilateral section to that in the contralateral section. Consistent with the results from in vivo images, the ratio in the sham groups was greater than 1, while the ratio in the MCAO groups was less than 1 (Fig. 5b, c and Additional file 1: Fig. S16c, d). In the MCAO group, the tracer mainly dispersed over the surface of the contralateral hemispheres, and the lower brightness indicated a lack of tracer distribution in the ipsilateral hemispheres.

The glymphatic system plays a significant role in clearing metabolic waste in the brain. We hypothesize that impaired glymphatic system function delays the clearance of metabolic waste, such as harmful proteins [47, 48]. To evaluate the clearance level after ischemia–reperfusion, we used BSA@IR-780 as a mimetic metabolic waste (Fig. 5d). BSA@IR-780 was infused into the brain at the coordinates of anterior/posterior (AP) −1.8 mm, medial/lateral (ML) −2.8 mm, and dorsal/ventral (DV) −1.8 mm from the bregma [48]. The deep tissue penetration of NIR-II fluorescence imaging allowed us to visualize the clearance efficiency by assessing the difference in fluorescence signal and fluorescence area (Fig. 5e). The normalized fluorescence signal showed that the sham group had lower intensity at the 6 h time point compared to the MCAO group demonstrating the sham group had a higher clearance efficiency than the MCAO group (Fig. 5f). The greater fluorescence area and higher brightness formation by residual NIR-II tracer BSA@IR-780 in harvested brain tissues and lower fluorescence signal in the liver further confirmed that ischemia–reperfusion injury damaged the clearance function of glymphatic system (Fig. 5g–i and Additional file 1: Fig. S16e). We also investigated the expression variation of AQP4, a water channel highly determined on CSF circulation along with the glymphatic pathway, using immunohistochemistry [49–51]. The results showed that ischemia–reperfusion impairment led to a significant reduction in AQP4 expression in the impaired area, which could be the main cause of impaired glymphatic influx and brain clearance (Fig. 5j, k).

## Conclusion

We propose the use of emerging NIR-II fluorescence imaging technology for glymphatic research, aiming to address the current limitations in glymphatic system imaging modalities, such as invasive imaging methods for slice tracer distribution analysis, limited field-of-view for two-photon imaging, low imaging quality for general microscope imaging, and expensive equipment for magnetic resonance imaging. The proposed NIR-II fluorescence imaging modality offers noninvasive, real-time, and high-resolution imaging advantages for dynamically studying the function of the glymphatic system. To achieve high-resolution images, we chose size-suitable nanoprobes and a signal collection window over 1200 nm. By using this 1200 nm window, we successfully obtained high-quality images of perivascular spaces located at the MCA. These high-quality images provide an opportunity for straightforward measurement of the size of PVS. By using this imaging technology, we determine the detailed interaction of glymphatic system function with anesthesia and cerebral ischemia–reperfusion injury. Overall, this proposed imaging technology is suitable for imaging the glymphatic function and has huge potential for probing the underlying mechanisms between the glymphatic system function and central nervous system diseases.

### Supplementary Information

**Additional file 1: Figure S1.** Using NIR-II fluorophore BSA@IR-780 as a CSF tracer. **Figure S2.** BSA@IR-780 enables imaging of CSF flow. **Figure S3.** Quantum dots (QDs) enables high-contrast imaging of CSF flow. **Figure S4. **QDs enable high-quality imaging through mimetic tissues intralipid. **Figure S5. **Collecting fluorescence signals over 1500 nm may hinder the accurate acquisition of signals compared to those collected over 1200 nm. **Figure S6.** Visualization of different doses of Dex for enhancing the glymphatic influx in the brain. **Figure S7. **Acquisition of in vitro images of the dorsal and ventral brain surfaces in the NIR-II window using different anesthetic mice. **Figure S8. **Comparing the intensity of in vitro dorsal and ventral brain surface harvested from different anesthetized mice at the 30 minute time point using selective anesthesia regimens. **Figure S9.** Evaluation of brain tracer distribution by slice imaging. **Figure S10.** NIR-II images acquired through intact skin demonstrate the impact of anesthetic regimens on the influx of CSF in CNS-draining lymph nodes. **Figure S11.** QDs as CSF tracers enable high-contrast imaging of the clearance of CSF through lymphatic vessels and LNs in the spine. **Figure S12.** In vivo NIR-II imaging of CSF efflux into mandibular lymph nodes under the doses of Dex 25 mg/kg and 0.015 mg/kg*2, respectively. **Figure S13.** Comparison of lymph node intensity. **Figure S14.** In vivo NIR-II imaging of CSF efflux into spines under different anesthesia regimens. **Figure S15.** NIR-II wide-field imaging allows for the collection of intensity data on whole-body vessel networks and the NIR-II tracer distribution in major organs under different anesthesia regimens. **Figure S16.** The suture-occluded method used to create the ischemia-reperfusion model.

## Data Availability

The datasets used and/or analyzed during the current study are available from the corresponding author on reasonable request.

## Abbreviations

CSF: Cerebrospinal fluid
Dex: Dexmedetomidine
ISO: Isoflurane
PVS: Perivascular spaces
MRI: Magnetic resonance imaging
mLNs: Mandibular lymph nodes
CM: Cisterna magna
QDs: Quantum dots
MCA: Middle cerebral artery
NIR-II: The second near-infrared window
AP: Anterior/Posterior
ML: Medial/Lateral
DV: Dorsal/Ventral
