# Supplementary material for: NIR-II nanoprobes for investigating the glymphatic system function under anesthesia and stroke injury
Source: J Nanobiotechnology. 2024 Apr 23;22:200. doi: 10.1186/s12951-024-02481-w (PMC11040925; doi:10.1186/s12951-024-02481-w)
Supplement: Supplementary file 1 — Additional file 1: Figure S1. Using NIR-II fluorophore BSA@IR-780 as a CSF tracer. Figure S2. BSA@IR-780 enables imaging of CSF flow. Figure S3. Quantum dots (QDs) enables high-contrast imaging of CSF flow. Figure S4. QDs enable high-quality imaging through mimetic tissues intralipid. Figure S5. Collecting fluorescence signals over 1500 nm may hinder the accurate acquisition of signals compared to those collected over 1200 nm. Figure S6. Visualization of different doses of Dex for enhancing the glymphatic influx in the brain. Figure S7. Acquisition of in vitro images of the dorsal and ventral brain surfaces in the NIR-II window using different anesthetic mice. Figure S8. Comparing the intensity of in vitro dorsal and ventral brain surface harvested from different anesthetized mice at the 30 minute time point using selective anesthesia regimens. Figure S9. Evaluation of brain tracer distribution by slice imaging. Figure S10. NIR-II images acquired through intact skin demonstrate the impact of anesthetic regimens on the influx of CSF in CNS-draining lymph nodes. Figure S11. QDs as CSF tracers enable high-contrast imaging of the clearance of CSF through lymphatic vessels and LNs in the spine. Figure S12. In vivo NIR-II imaging of CSF efflux into mandibular lymph nodes under the doses of Dex 25 mg/kg and 0.015 mg/kg*2, respectively. Figure S13. Comparison of lymph node intensity. Figure S14. In vivo NIR-II imaging of CSF efflux into spines under different anesthesia regimens. Figure S15. NIR-II wide-field imaging allows for the collection of intensity data on whole-body vessel networks and the NIR-II tracer distribution in major organs under different anesthesia regimens. Figure S16. The suture-occluded method used to create the ischemia-reperfusion model. [file 12951_2024_2481_MOESM1_ESM.docx]

Additional file

NIR-II nanoprobes for investigating the glymphatic system function under anesthesia and stroke injury

Bin Sun^1,2^, Danlan Fang^1,2^, Wenzhong Li^1^, Mengfei Li^1^ and Shoujun Zhu^1,2^*

^1^Joint Laboratory of Opto-Functional Theranostics in Medicine and Chemistry, First Hospital of Jilin University, Changchun, 130021, China

Email: [sjzhu@jlu.edu.cn](mailto:sjzhu@jlu.edu.cn)

^2^State Key Laboratory of Supramolecular Structure and Materials, Center for Supramolecular Chemical Biology, College of Chemistry, Jilin University, Changchun 130012, China

Materials and methods…………………………………………………….....Pages 3-10

Additional file figures……………………………………………………..…Pages 11-22

**Materials and methods**

**Materials**

PbCl_2_, CdO, Na_2_CO_3_, *N*,*N*′-dicyclohexylcarbodiimide (DCC), poly(acrylic acid) (*M*W = ∼1800), hexane, toluene, dimethyl formamide (DMF) and trichloromethane were purchased from Aladdin. Sulfur powder was purchased from Alfa. Oleylamine, oleic acid, 1-octadecene (ODE), and 2-(Nmorpholino) ethanesulfonic acid (MES) hydrate, 1-Ethyl-3-(3-dimethyllaminopropyl) carbodiimide hydrochloride (EDC) were purchased from Sigma-Aldrich. The mPEG-NH_2_ (average molecular weight: 5,000) and 8Arm-PEG-NH_2_ (average molecular weight: 40,000) were purchased from Xi’an Ruixi Biological Technology Co., Ltd. Amicon ultra-15 (100 kDs) was purchased from Sigma-Aldrich. Bovine serum albumin and cyanine dye were purchased from Sigma-Aldrich. ICG was purchased from TCI (Shanghai) Development Co., Ltd. The artificial cerebrospinal fluid (aCSF) was purchased from Beijing Solarbio Technology Co., Ltd. Isoflurane (ISO) was purchased from RWD Life Science Co., Ltd. Dexmedetomidine powder was purchased from Aladdin. Avertin (also known as 2,2,2-tribromoethanel) was purchased from Nanjing Aibei Biotechnology Co., Ltd.

**Characterization**

Transmission electron microscope (JEM-2100F) was used to evaluate the morphology of QDs. The formation of BSA@IR-780 complex was analyzed using gel electrophoresis, followed by imaging the gel in the NIR-II window.

**Synthesis of NIR-II Fluorophores**

**Synthesis of quantum dots (PbS@CdS).** The method for synthesizing PbS@CdS was based on previous reports with some modifications [1, 2]. The sulfur precursor solution was prepared by mixing 0.08 g (5 mmol) of sulfur powder and 7.5 mL of oleylamine in a two-neck flask at 120 °C. The mixture was degassed using a vacuum pump for 30 minutes and protected with nitrogen until ready for use. The lead precursor solution was prepared by mixing 0.834 g (3 mmol) of PbCl_2_ with 7.5 mL of oleylamine in a three-neck flask. The mixture was degassed using a vacuum pump for 90 minutes at 120 °C and then protected with nitrogen. The temperature was increased to 160 °C under nitrogen protection and maintained for 10 minutes. Next, 2.25 mL of the sulfur precursor solution (0.75 mmol of S) was rapidly injected into the Pb precursor solution (3 mmol of Pb) under stirring. The temperature was maintained at 160 °C throughout the reaction for 30 minutes. The reaction was quenched by adding 10 mL of cold hexane and 20 mL of ethanol. The products were collected by centrifugation at 8000 rpm at 10 °C and the precipitate was re-suspended in 10 mL of hexane. This centrifugation step was repeated three times. Afterwards, the precipitation was washed with oleic acid until the supernatant became colorless. The PbS QDs were collected and re-suspended in 15 mL of 1-octadecene. PbS@CdS QDs were synthesized using the cation-exchange procedure. CdO (1.2 g, 9.2 mmol), oleic acid (8 mL), and ODE (20 mL) were mixed in a three-neck flask and degassed using a vacuum pump for 30 minutes at 90 °C. The solution was then heated to 200 °C under nitrogen protection and maintained at 200 °C for 120 minutes. It was then cooled down to 100 °C. Once the temperature stabilized at 100 °C, 5 mL of the previously prepared PbS QDs suspended in ODE was bubbled with argon for 10 minutes and then rapidly injected into the Cd precursor. The reaction was maintained at 100 °C for 20 minutes and quenched with 5 mL of cold hexane. PbS@CdS QDs were precipitated with ethanol and then re-dispersed in hexane.

**Synthesis of oleylaminepoly(acrylic acid (OPA).** To enhance the water solubility of PbS@CdS QDs, we utilized an amphiphilic polymer called oleylaminepolyrylic acid (OPA) for the modification of QDs. The synthesis of OPA followed a previously established method [1, 3]. In a round-bottom flask, 0.9 g of polyacrylic acid powder (average MW = ~1800) and 1.56 g of DCC were mixed together, and then 10 mL of DMF was added to dissolve the mixture. Under stirring, approximately 1.2 mL of oleylamine was added dropwise to the solution. The molar ratio of oleylamine to poly(acrylic acid) was 30%. The solution was stirred for 16 hours. Then, 50 mL of 0.5 M HCl was added to the reaction solution. The precipitate was separated by centrifugation and re-dissolved in 3 mL of methanol solution. Next, 20 mL of 1 M HCl was added to the methanol solution. The mixture was centrifuged and the precipitate was collected. This procedure was repeated at least 5 times. The collected precipitate was dissolved in 5 mL of chloroform and washed with 10 mL of 1 M HCl. The organic phase was collected and dried overnight using anhydrous Na_2_SO_4_. The chloroform was then removed under vacuum, and the resulting white solid was collected and stored at 4°C.

**Coating the PbS@CdS QDs with amphiphilic polymer OPA.** PbS@CdS QDs (5.0 mg) were dissolved in 1.0 mL of chloroform. Additionally, 15 mg of OPA was dissolved in 1.0 mL of chloroform. The chloroform solution containing OPA was then slowly added dropwise to the solution containing PbS@CdS QDs. The system was stirred at 600 rpm at room temperature for 30 minutes. The chloroform was subsequently removed under vacuum using a rotary evaporator. The QDs-OPA complex was then dissolved in 2 mL of a 50 mM Na_2_CO_3_ solution through overnight sonication. The QDs-OPA complex was collected using an ultracentrifuge at 50,000 rpm for 1 hour. Finally, the resulting QDs-OPA complex was dissolved in pH 8.5 MES buffer (0.01 M) and stored at 4 °C for further modification.

**Conjugation of the QDs-OPA with highly water-soluble polyethylene glycol (PEG).** OPA-modified QDs (5 mg) were dissolved in 1 mL of pH 8.5 MES buffer (0.01 M). Then, 15 mg of mPEG-amine (MW ~ 5K) and 5 mg of 8-Arm PEG-amine (MW ~ 40 K) with a molar ratio of 24:1 were dissolved in 0.5 mL of MES and mixed with the QDs solution. Next, 10 mg of EDC was dissolved in 0.5 mL of pH 8.5 MES and added dropwise to the mixed solution while stirring at 600 rpm. The solution was stirred at room temperature overnight. The PEGylated QDs were purified using a 100 kDa filter and washed five times with 1x PBS to remove any excess reactants. The purified product was then dissolved in aCSF and stored at 4 °C for use as a CSF tracer.

**Synthesis of BSA@IR-780 CSF tracer.** The method for synthesizing BSA@IR-780 is based on our previous reports [4, 5]. BSA protein, used as an enhancer to improve fluorescence intensity and photostability of cyanine dyes, was dissolved in 1 × PBS at a concentration of 600 μM. The cyanine dye (IR-780) was selected due to its optimal combination with BSA and was dissolved in dimethyl sulfoxide (DMSO) at a concentration of 20 mM. For a typical reaction, 15 μL of 20 mM IR-780 and 485 μL of 1 × PBS were added to 500 μL of 600 μM BSA solution. The system was vortexed for 10 seconds and heated at 50 °C on a shaker for 2 hours. The solution was then converted from 1 × PBS to aCSF using an Amicon Centrifugal Filter with a 30 kDa cutoff.

**Animals.** Male C57BL/6 mice aged 8-10 weeks, weighing between 21 and 25 g, were obtained from Liaoning Changsheng Biotechnology Co. Ltd. The mice were housed in a 12-hour light/12-hour dark cycle, with the lights on at 7:00 a.m. and off at 7:00 p.m. They had unrestricted access to bedding, nesting materials, food, and water. Ambient temperature was constantly controlled at 24°C and the ambient humidity was controlled at 30-70%. All animal experiments were conducted in accordance with the institutional guidelines and were approved by the Animal Ethical Committee of The First Hospital of Jilin University (protocol number: 20210642). All efforts were made to keep animal usage to a minimum.

**Construction of the NIR-II fluorescence imaging system.** For more detailed procedures on constructing the NIR-II imaging equipment, readers can refer to these classical literature sources [6-8]. In this study, a two-dimensional InGaAs array (Raptor Photonics) was used to collect the NIR-II fluorescence signal. All imaging experiments were conducted using an 808 nm laser, with a power density of approximately 0.065 W/cm^2^, and passed through an 850 short-pass filter. The emitted luminescence from QDs was filtered using a combination of long-pass filters at 900 nm and 1200 nm, while the emitted luminescence from BSA@IR-780 was filtered using a combination of filters at 1000 nm and 1100 nm. These filters were used to ensure that the Raptor camera collected signals within the NIR-II region. A short-wave infrared lens (75 mm focal length or 50 mm focal length) was installed on the InGaAs detector to focus on the required image region, with a field of view of ~1.9 x 1.5 cm^2^ or with a field of view of ~2.4 x 1.9 cm^2^. The image region was consistently positioned in the middle region of the laser coverage throughout all imaging experiments. The exposure time for capturing images was adjusted using computer controls and is described in the corresponding legend.

**Photostability measurement of QDs and BSA@IR-780 tracers.** The clinically-used ICG and QDs were prepared separately in PBS solutions. They were then exposed to continuous laser irradiation at a power density of approximately 0.065 W/cm^2^. The images were collected during continuous laser irradiation. The photostability measurement for BSA@IR-780 and free IR-780 was conducted following the same experimental procedure. 0.005 W/cm^2^ was used to replace 0.065 W/cm^2^ for measurement the photostability of BSA@IR-780.

**Using intralipid for testing the penetration depth.** To test the resolution of wavelength-dependent images, we immersed capillary tubes filled with QDs or ICG in PBS solutions into a 100% intralipid solution at a depth of 2 mm. The fluorescence signal was collected in three different windows: 900‒1000 nm, 1000–1100 nm, and over 1200 nm. A laser intensity of 65 mW/cm^2^ was used to excite the fluorophores, and the exposure times were adjusted to ensure similar fluorescence signal collection in each image. To measure the penetration performance of QDs or ICG, we covered the capillary tube filled with the fluorophore solution with intralipid at different depths ranging from 0 mm to 6 mm. The image acquisition conditions were the same as those in the wavelength-dependence images resolution experiment.

**Infusion of NIR-II tracer into the cisterna magna (CM).** The mice were secured in a stereotaxic frame while under ISO anesthesia. Subsequently, the CM was exposed. A PE10 tube filled with NIR-II tracer was inserted into the CM through a 30 gauge needle bridge. The tracer, either QDs or BSA@IR-780, was dissolved in an aCSF solution at a concentration of 5 mg/mL or 300 μM. The inserted PE10 tube was first sealed with 3M vetbond tissue adhesive and then completely sealed with dental cement. The tracer was delivered using a syringe pump (Harvard Apparatus) at a rate of 1 μL/min for 7 minutes, with a total volume of 7 μL. The body temperature was maintained at 37 ℃ using a heating pad. The experimental procedure for evaluating the effect of dexmedetomidine (Dex) on the glymphatic system is described in the following content.

**Acquisition of imaging beyond 1200 nm enables high-resolution imaging of the CSF movement/glymphatic system through the intact scalp.** The hair was shaved prior to the commencement of the imaging experiment. The mice were positioned in a prone pose under the imaging setup, and the brain was imaged within a field of view measuring ~1.9 x 1.5 cm^2^. A wide-view image (~1.9 x 1.5 cm^2^) was obtained using a specific lens with a focal length of 75 mm. The imaging window (900–1000 nm, 1000–1100 nm, and over 1200 nm) was utilized to capture the fluorescence signal emitted by QDs through the intact scalp or removal of scalp.

The imaging window (900–1000 nm, 1000–1100 nm, and over 1100 nm) was utilized to capture the fluorescence signal emitted by BSA@IR-780 through the intact scalp or removal of scalp. and the brain was imaged within a field of view measuring ~2.4 x 1.9 cm^2^. A wide-view image (2.4 x 1.9 cm^2^) was obtained using a specific lens with a focal length of 50 mm.

The imaging quality was assessed by quantitatively analyzing the SBR.

**Visualization of the NIR-II tracer influx into brain.** The brain was focused on the field view of ~1.9 x 1.5 cm^2^. The surgery and sealing procedures were performed under ISO anesthesia. Different doses of Dex were intraperitoneally injected to supplement the ISO anesthesia before the start of infusion. For the 0.2 mg/kg and 25 mg/kg groups, Dex was intraperitoneally injected 5 minutes before the start of infusion. For the 0.015 mg/kg*2 group, the mice received twice injection at 30 minute and 5 minute respectively before the start of infusion. The distribution was monitored for 30 minutes with an interval of 5 minutes, and the first image (0 minutes) was acquired at the start of infusion. After allowing the NIR-II tracer to circulate for 30 minutes, the brain was collected and the tracer distribution on the dorsal and ventral brain surfaces was imaged. The imaging conditions were 100 ms, 1200 LP, and 65 mW/cm^2^ power density.

**Visualization of the NIR-II tracer drainage into lymph nodes**. The hair was shaved before the imaging experiment to expose the neck skin. The infusion was performed with the mice in a prone position. After the infusion was completed, the mice were placed in a supine position for imaging of the lymph node. The first image was acquired 10 minutes after the start of the infusion, and the movement of the tracer was monitored for 60 minutes with intervals of 5 min. The imaging conditions were 100 ms or 50 ms, 1200 LP, and 65 mW/cm^2^ power density.

**Visualization of the NIR-II tracer distribution in spine**. The hair was shaved prior to the imaging experiment. The mice were placed in a prone position within a field of view measuring ~8.7 x 7.7 cm^2^, and the infusion was performed using a similar procedure as described above. A wide-view image (~8.7 x 7.7 cm^2^) was obtained using a specific lens with a focal length of 35 mm. The distribution of the tracer in the spine was monitored for 90 minutes after the start of infusion, with intervals of 5 minutes. After monitoring the tracer distribution in the spine, the vessel network in lateral and supine positions was imaged. The imaging conditions for visualizing the vessel network were set at 100 ms or 2 ms 1200 LP, and 65 mW/cm^2^.

**Note:** To avoid the circadian control of glymphatic system function, the imaging experiments were primarily conducted between ZT4 and ZT10.

**Transient middle cerebral occlusion (tMCAO).** The ischemia-reperfusion model (tMCAO) was used to evaluate the function of the glymphatic system after a stroke. The mice were placed on a heating pad to maintain their body temperature at 37 ℃. An incision was made to expose the common carotid artery (CCA), internal carotid artery (ICA), and external carotid artery (ECA). Surgical sutures were then used to ligate the CCA and ECA (distal end). The ICA was occluded using a bulldog clamp. A small incision was made in the proximal side of the ECA, allowing a filament to be inserted into the ECA and subsequently turned into the CCA to block blood flow into the MCA. After 60 minutes of occlusion, the filament was withdrawn to allow reperfusion. The sham mice underwent the same surgical procedure, but without the insertion of the filament.

**The NIR-II tracer BSA@IR-780 distribution in coronal brain section.** The brain was removed 30 minutes after the start of the infusion of NIR-II tracer BSA@IR-780 into the central nervous system. The brain was then fixed with 4% paraformaldehyde (PFA) for 12 h. The brain slice, which was 100 μm thick, was prepared using a vibratome. A total of twelve slices were collected at 300-μm intervals, starting 1.6 mm anterior to the bregma, in order to calculate the mean total tracer influx for the entire brain. The brain slices were then imaged using a scanning imaging system (Azurespot).

**Triphenyltetrazolium chloride staining.** A 2% commercial triphenyltetrazolium chloride (TTC) solution was used to stain the entire brain and brain sections after stroke. Stroke mice and sham mice were anesthetized with avertin (200 mg/kg) and transcardially perfused with 20 mL of pre-cooled PBS. The whole brain was immediately harvested and immersed in preheated TTC solution (37 ℃). The brain in TTC solution was then placed in 37 ℃ oven for 30 minutes, with shaking every 5 minutes. For brain section staining, the whole brain was frozen at -20 ℃ for 30 minutes, and then 1 mm thick brain sections were prepared. These brain sections were immersed in preheated TTC solution (37 ℃) and stained in 37 ℃ oven for 20 minutes. After staining, the whole brain and brain sections were fixed with 4% paraformaldehyde (PFA) for 10 minutes. The stained whole brain and brain sections were imaged.

**Visualization of glymphatic system function after tMCAO.** After 24 hours of reperfusion, the NIR-II tracer was injected into the CM and its distribution was monitored for 30 minutes following the start of infusion.

**In vivo imaging of the impaired parenchymal clearance after tMCAO.** After 24 hours of reperfusion, the mouse was anesthetized with ISO and the scalp was removed to expose the skull. A hole was created at coordinates AP: -1.8 mm from Bregma, LM: −2.8 mm, and DV: −1.8 mm. The NIR-II tracer BSA@IR-780 was then infused into the parenchyma at a rate of 0.5 μL/min for 4 minutes. After the injection, the mice recovered and were allowed to move freely. The images were collected after infusion for 6 hours.

**Imaging analysis.** All image processing and fluorescence intensity quantification were performed using Fiji software (version 1.8.0, National Institutes of Health, USA). Graphs were created using GraphPad Prism (version 8.0.2, GraphPad Software) and Origin (version 2019b, Origin Software). The procedure for processing images of NIR-II tracer influx into the brain is as follows. The images, initially in 16-bit format, collected within a 30-minute circulation period, underwent background subtraction by subtracting the images taken at the 0-minute time point. After that, the images were adjusted using a brightness threshold of 0 to 20000 and converted to 8-bit format. Three regions of interest (ROI) were manually drawn around the brain region, left MCA region, and right MCA region, respectively. The mean intensity of each ROI was then calculated. Quantifying the tracer distribution within the lymph node and spine: the images were adjusted using a brightness threshold ranging from 0 to 65535 and converted to an 8-bit format; the ROI was manually drawn and used to calculate the mean intensity.

**Note:** The ROI was manually delineated based on the outline displayed in the brightest images.

Quantification of tracer distribution in coronal brain sections involved manually drawing the outline of each coronal brain slice and calculating the mean intensity within each slice. The total intensity of twelve slices was divided by twelve to obtain the intensity value per mouse.

**Statistical analysis.** All statistical analyses were conducted using GraphPad Prism8. The data in all graphs are presented as mean ± standard error of the mean (SEM) over the individual data points and lines from each mouse. T test and ANOVA test were selected and are described in the figure legends. A *P* value <0.05 was considered statistically significant.

**Additional file figures**
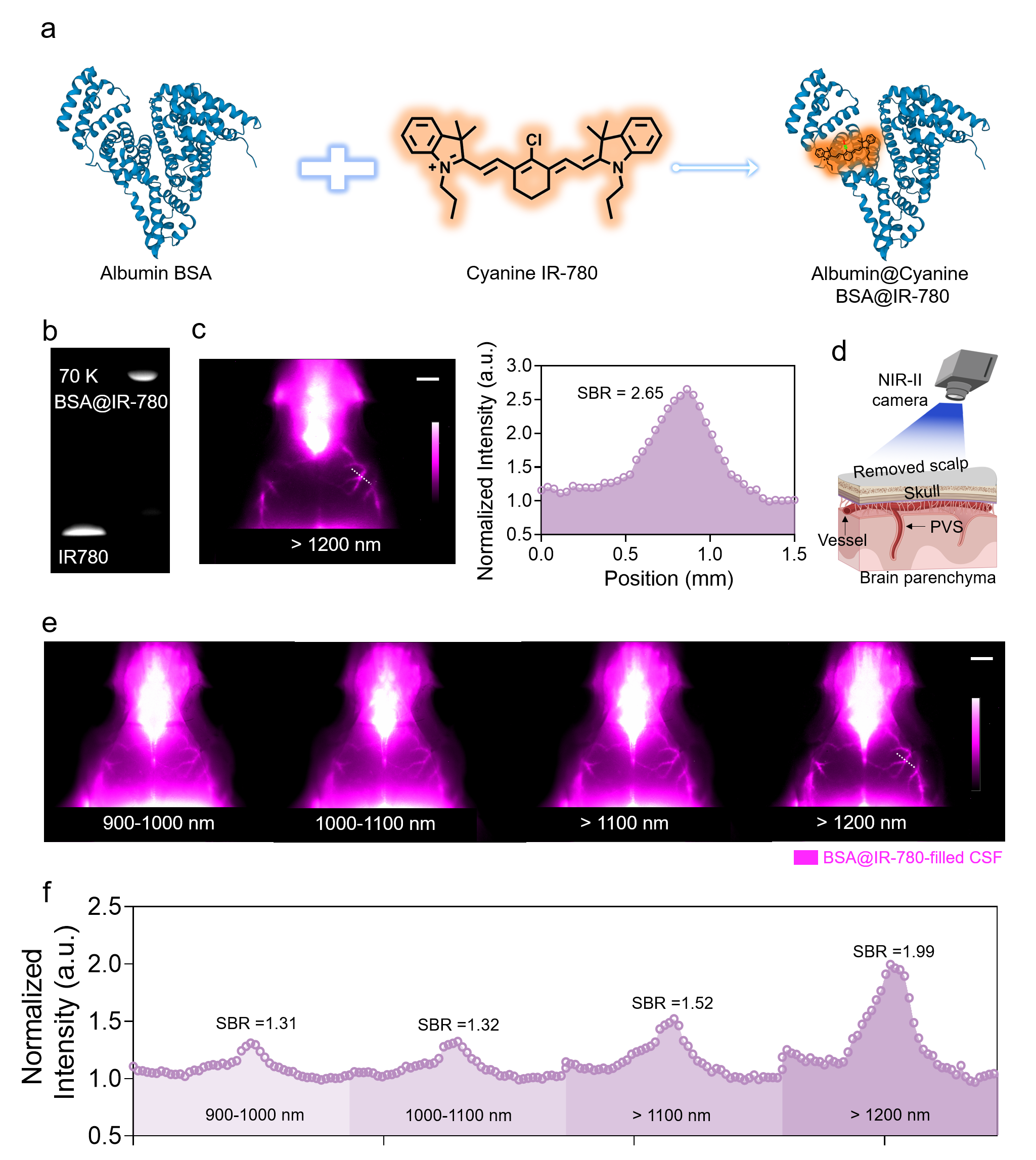


Figure S1. Using NIR-II fluorophore BSA@IR-780 as a CSF tracer. a) Preparation of BSA@IR-780. b) Analysis of BSA@IR-780 by gel electrophoresis. c) BSA@IR-780 imaging of the MCA over 1200 nm through the intact skull. d) The cartoon shows the collection of images with the removal of the scalp. e) Acquisition of images through 900-1000 nm, 1000-1100 nm, over 1100 nm, and over 1200 nm with the removal of the scalp. f) Quantification of the corresponding SBR values in e). White scale bar: 2 mm. The cross-sectional fluorescence signal along the white dish inserted in e was collected to quantify the SBR.


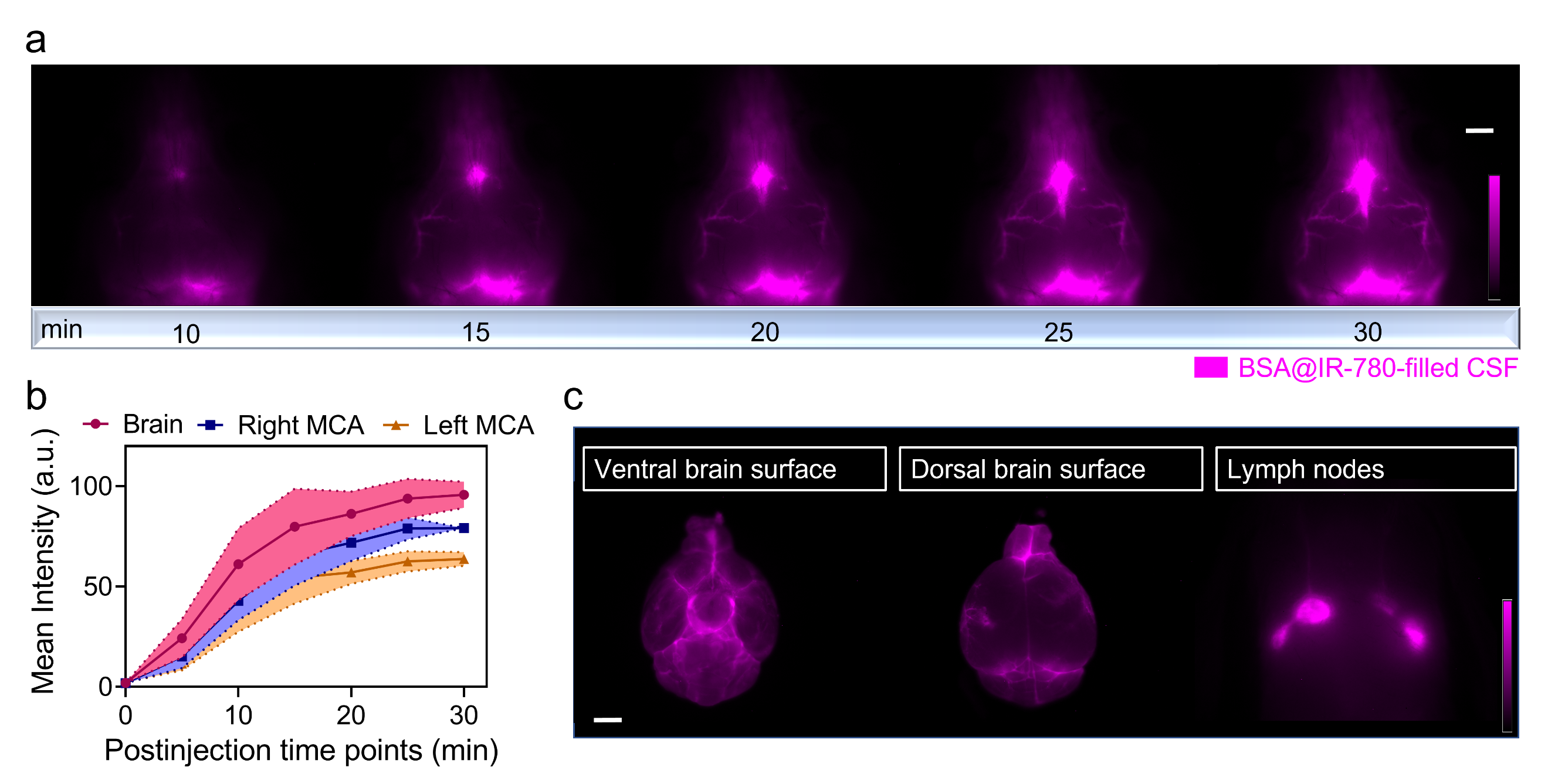


Figure S2. BSA@IR-780 enables imaging of CSF flow. a) BSA@IR-780 images CSF flow along the PVS after CM injection. b) Quantification of fluorescence signal in selective ROI regions. c) Collection of brightness signal from in vitro brain and in vivo LNs suggests that BSA@IR-780 has the ability to image the glymphatic system in rodent model. White scale bar: 2 mm.


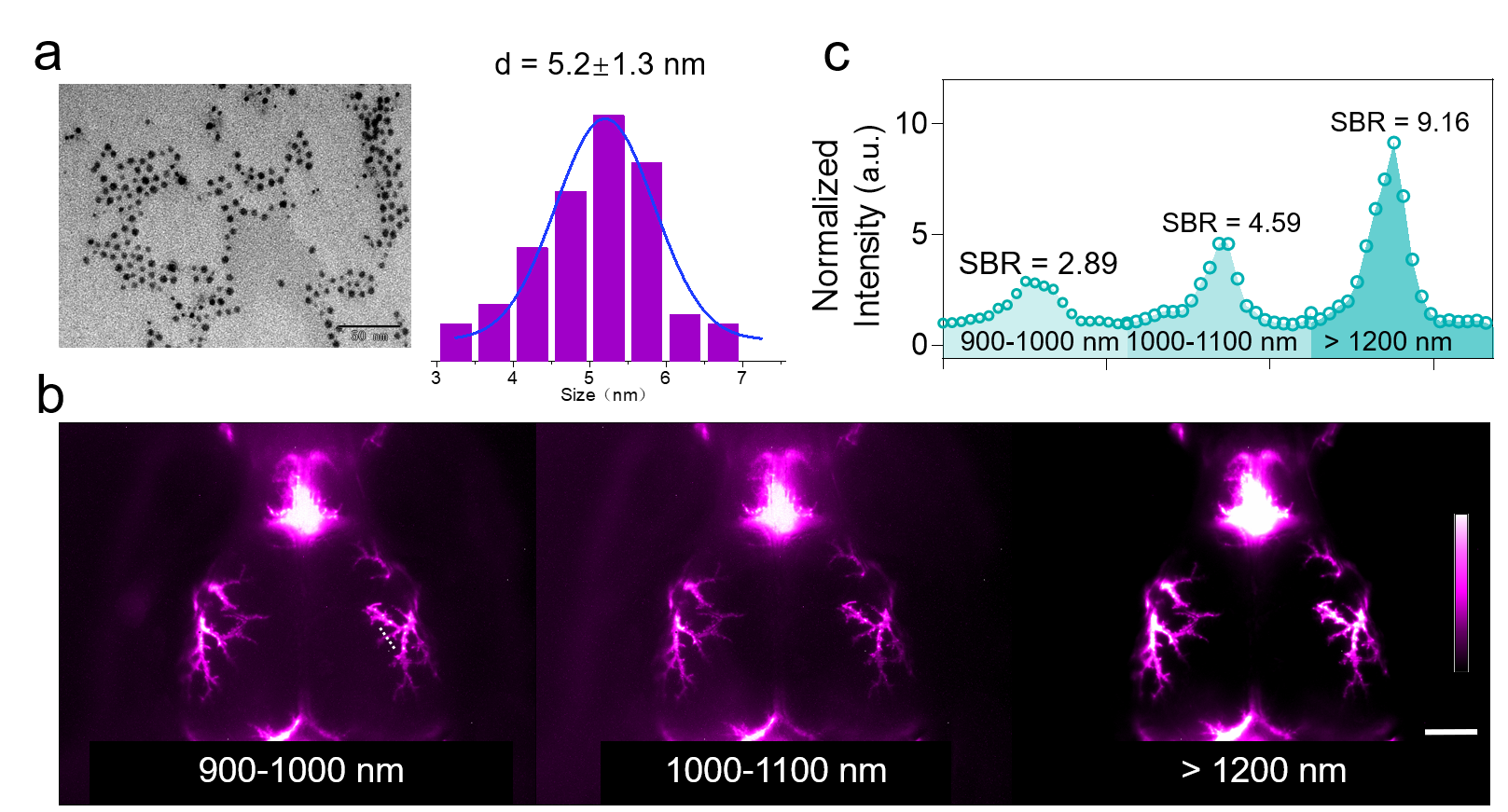


Figure S3. Quantum dots (QDs) enables high-contrast imaging of CSF flow. a) Transmission electron microscopy (TEM) imaging of QDs. b) In vivo high-contrast imaging of CSF flow with scalp removal by CM-injection of QDs. c) SBR of in vivo CSF images obtained in b. The SBR was quantified by collecting the cross-sectional fluorescence signal along the white dish inserted in b. White scale bar: 2 mm.


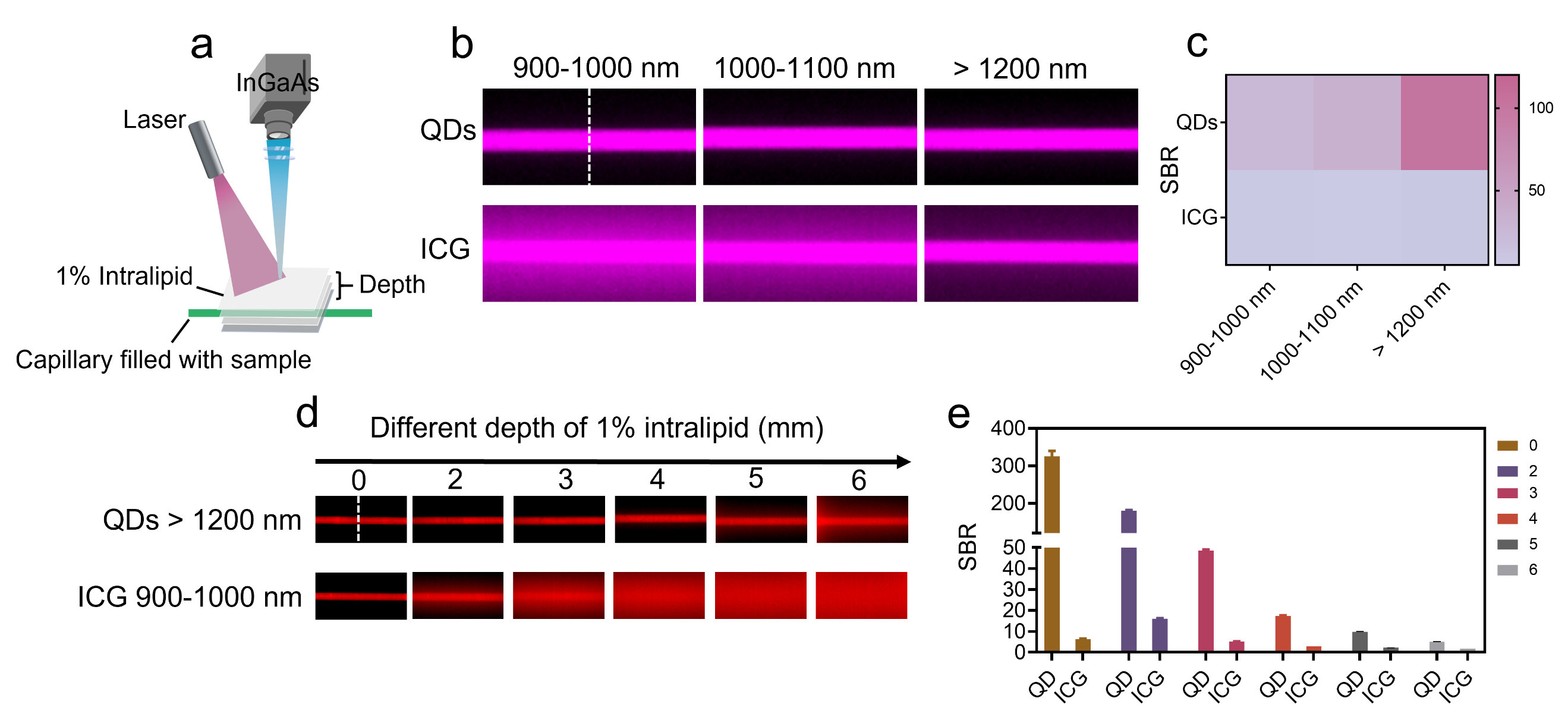


Figure S4. QDs enable high-quality imaging through mimetic tissues intralipid. a) Schematic of penetration depth imaging experiments of QDs and ICG solution in artificial CSF. b) Capillary tubes filled with QDs solution or ICG solution were immersed in a 2 mm depth of 1% intralipid solution, and the signals were collected in different sub-NIR-II windows (900-1000 nm, 1000-1100 nm, and over 1200 nm). c) The corresponding SBR for QDs and ICG quantified from the values along the white dish in b. d) NIR-II images of QDs (signal collection over 1200 nm) and ICG (signal collection within 900-1000 nm) at different depths. e) The corresponding SBR for QDs and ICG quantified from the values along the white dish in d.

**Data Note.** The capillary tube filled with QDs showed a clearer outline in all sub-NIR-II windows compared to ICG, indicating that the QDs were effective as a contrast agent for probing biological information in deep tissues. The highest SBR was observed for QDs over 1200 nm. It is worth noting that previous reports using QDs for imaging the whole-body blood circulation system or tumors through tail-vein injection typically filtered the emitting signal with a 1500-nm filter. In this study, the fluorescence signal was collected over 1200 nm due to the following reasons: ⅰ) Only a few microliters of QDs (7 µL) were injected into the CM, which is much less than the dose used in tail-vein injection (200 µL) and may result in inadequate whole body signal under the 1500 nm filter. ⅱ) We found that using a 1500 nm filter hindered accurate signal collection during the imaging experiment (Figure S5, Supporting Information). Overall, the 1200 nm long-pass filter was suitable for assessing CSF flow.


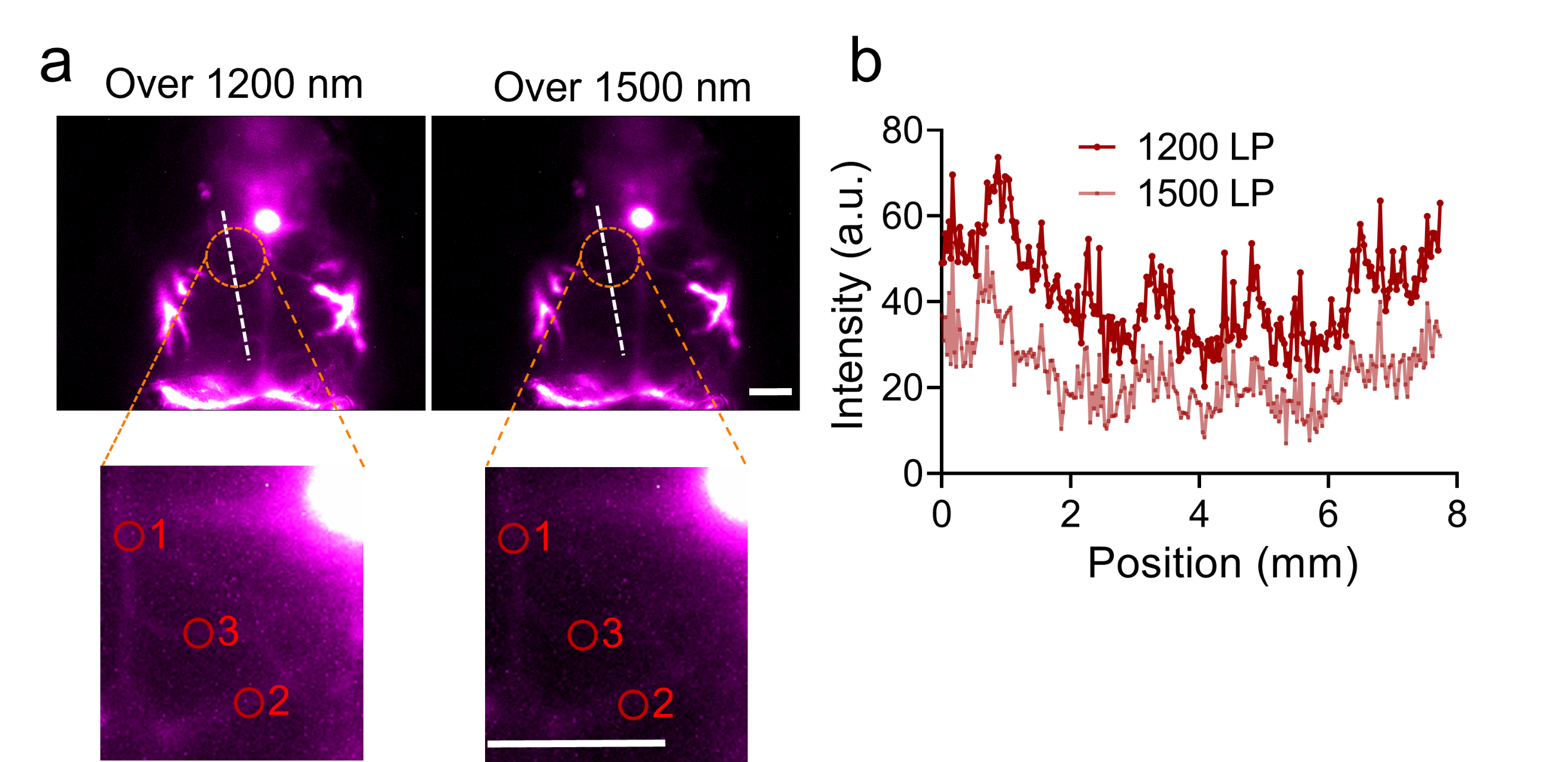


Figure S5. Collecting fluorescence signals over 1500 nm may hinder the accurate acquisition of signals compared to those collected over 1200 nm. White scale bar: 2 mm.


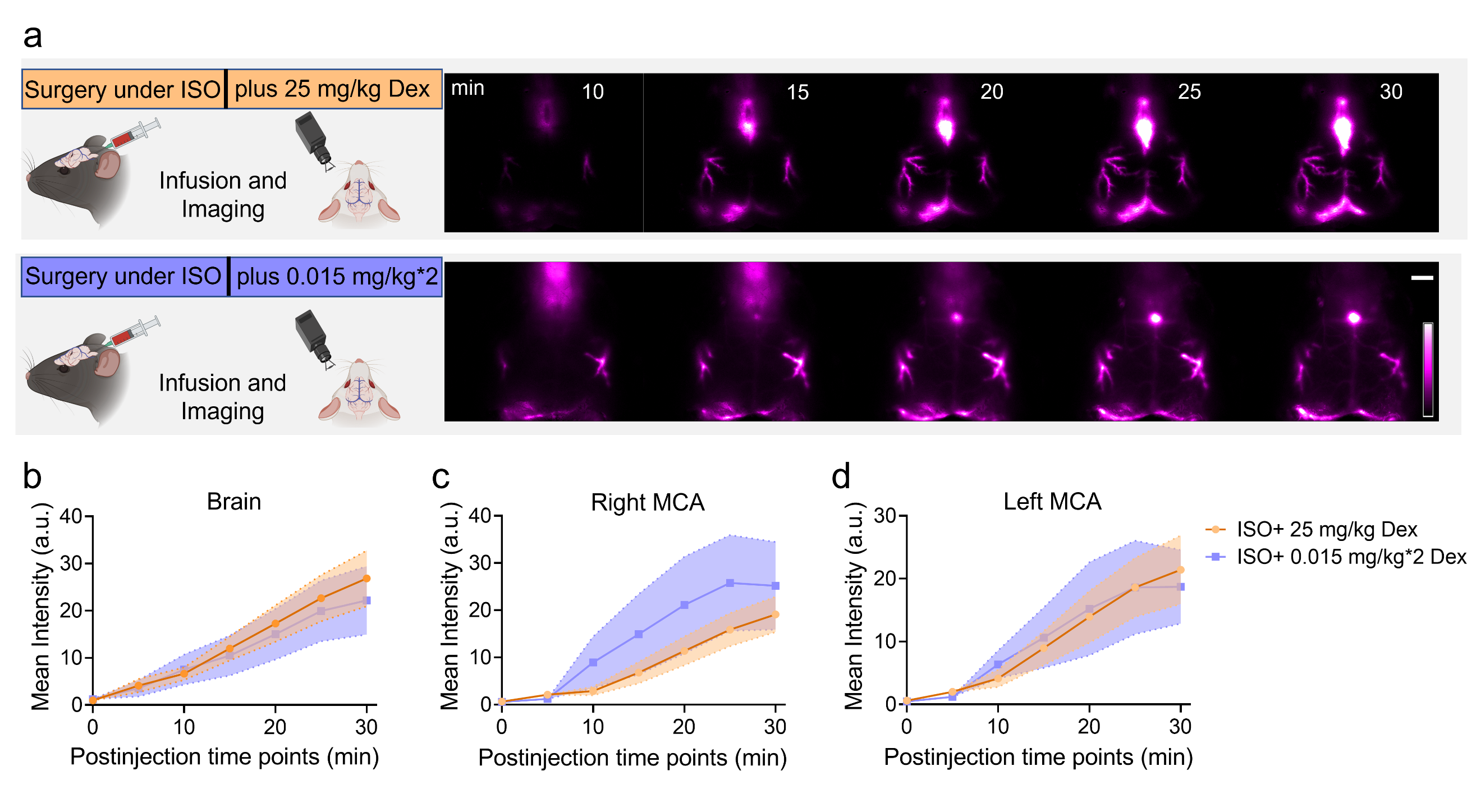


Figure S6. Visualization of different doses of Dex for enhancing the glymphatic influx in the brain. a) In vivo imaging of the CSF influx in the brain under the doses of Dex 25 mg/kg and 0.015 mg/kg*2. Mean intensity of the b) brain, c) right MCA, and d) left MCA over 30 minutes after the beginning of CM injection. n=7 for ISO plus Dex 25 mg/kg group, n=5 for ISO plus Dex 0.015 mg/kg*2 group. White scale bar: 2 mm.


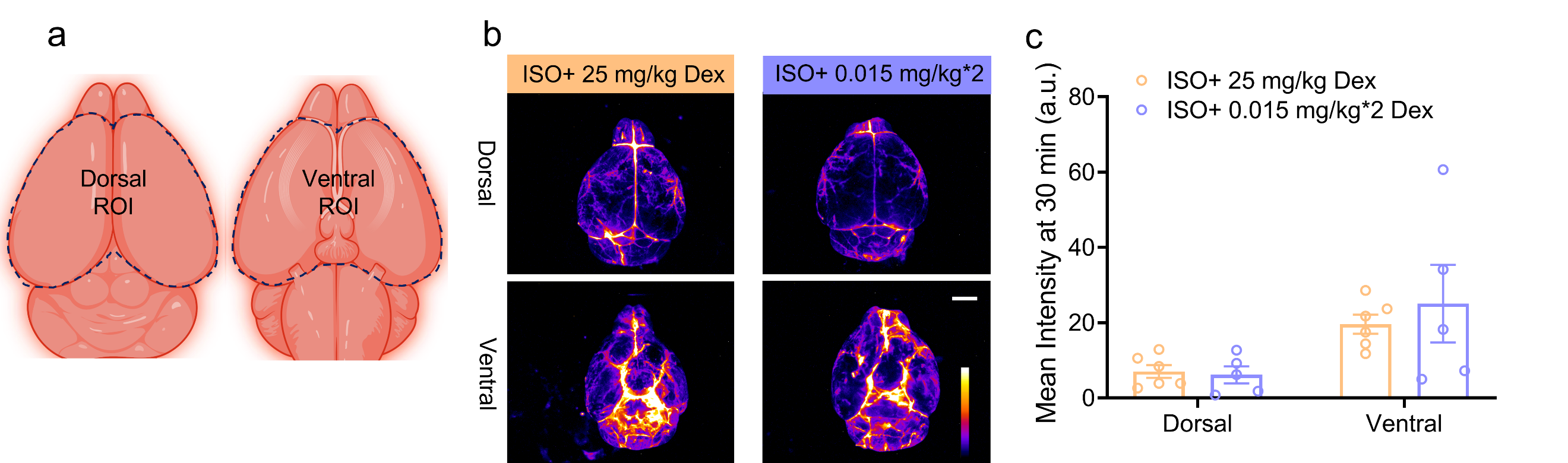


Figure S7. Acquisition of in vitro images of the dorsal and ventral brain surfaces in the NIR-II window using different anesthetic mice. a) A cartoon displaying the region for collecting the fluorescence signal from the dorsal and ventral brain tissue surfaces. b) Representative images showing the distribution of NIR-II tracer on the brain tissue surfaces under the doses of Dex 25 mg/kg and 0.015 mg/kg*2. c) Quantification of the corresponding fluorescence signals in b. n=6 for ISO plus Dex 25 mg/kg group, n=5 for ISO plus Dex 0.015 mg/kg*2 group. White scale bar: 2 mm.


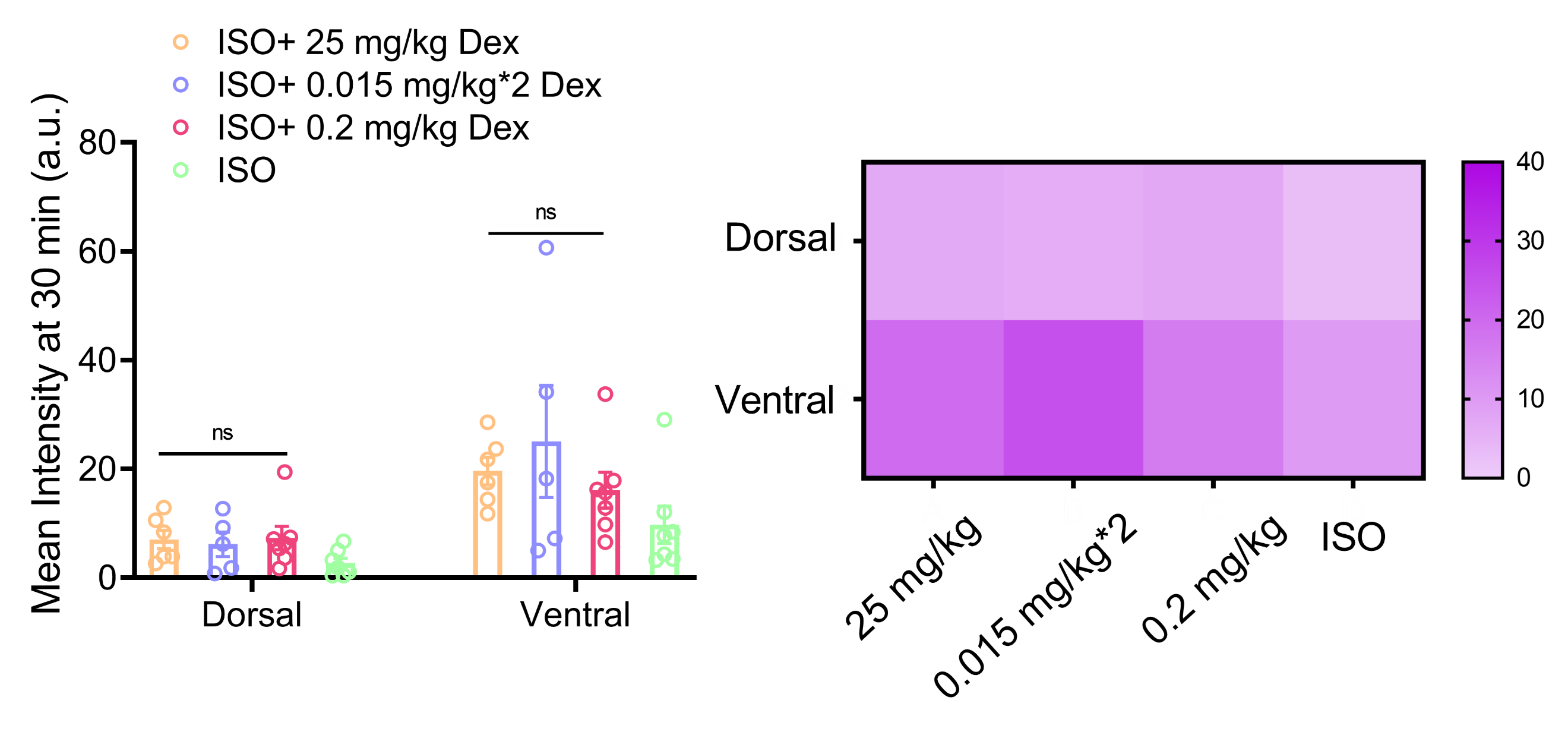


Figure S8. Comparing the intensity of in vitro dorsal and ventral brain surface harvested from different anesthetized mice at the 30 minute time point using selective anesthesia regimens. n=6 for ISO plus Dex 25 mg/kg group, n=5 for ISO plus Dex 0.015 mg/kg*2 group, n=7 for ISO plus Dex 0.2 mg/kg group, n=7 for ISO group. To facilitate a direct comparison of the effects of different anesthesia regimens, the data points for the ISO alone group and the ISO plus Dex 0.2 mg/kg group are displayed again from Figure 2i, j.


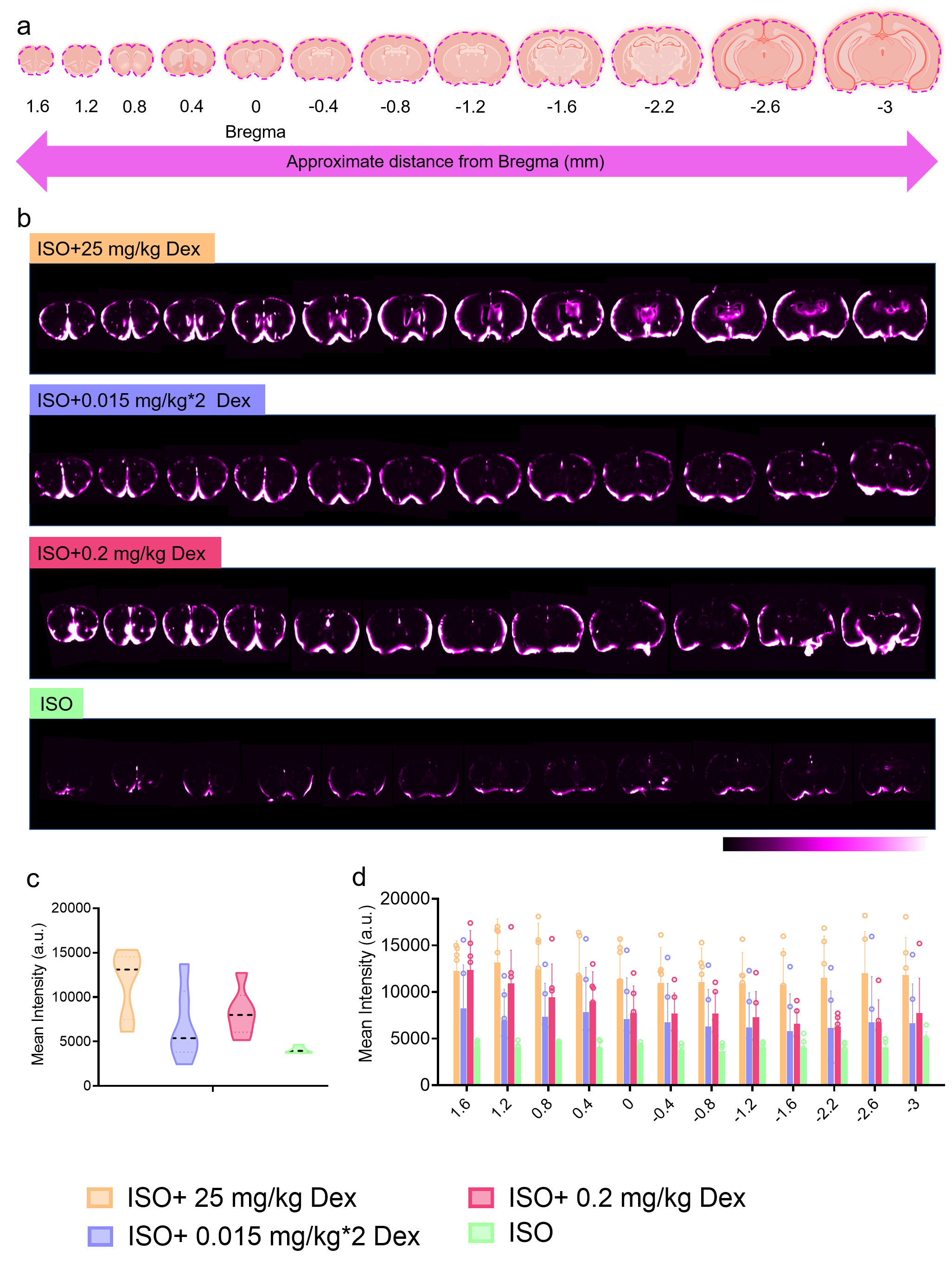


Figure S9. Evaluation of brain tracer distribution by slice imaging. a) The diagram illustrates the estimated distance from bregma for collecting the slices to capture images of NIR-II tracer distribution in coronary brain slices. Twelve slices were collected to calculate the average intensity per mouse. b) Representative images of slices for all groups of anesthetized mice. c) The average intensity across all brain slices in all groups of anesthetized mice. d) The anterior/posterior distribution of BSA@IR-780 in all groups of anesthetized mice. n=6 per group. To facilitate a direct comparison, the data points for the ISO alone group and the ISO plus Dex 0.2 mg/kg group in c are displayed again from Figure 2m.

**Data Note.** The slices imaging results also showed that mice anesthetized with 25 mg/kg exhibited the highest mean intensity, while those anesthetized with 0.2 mg/kg had medium intensity, and those anesthetized with 0.015 mg/kg*2 had the lowest intensity.


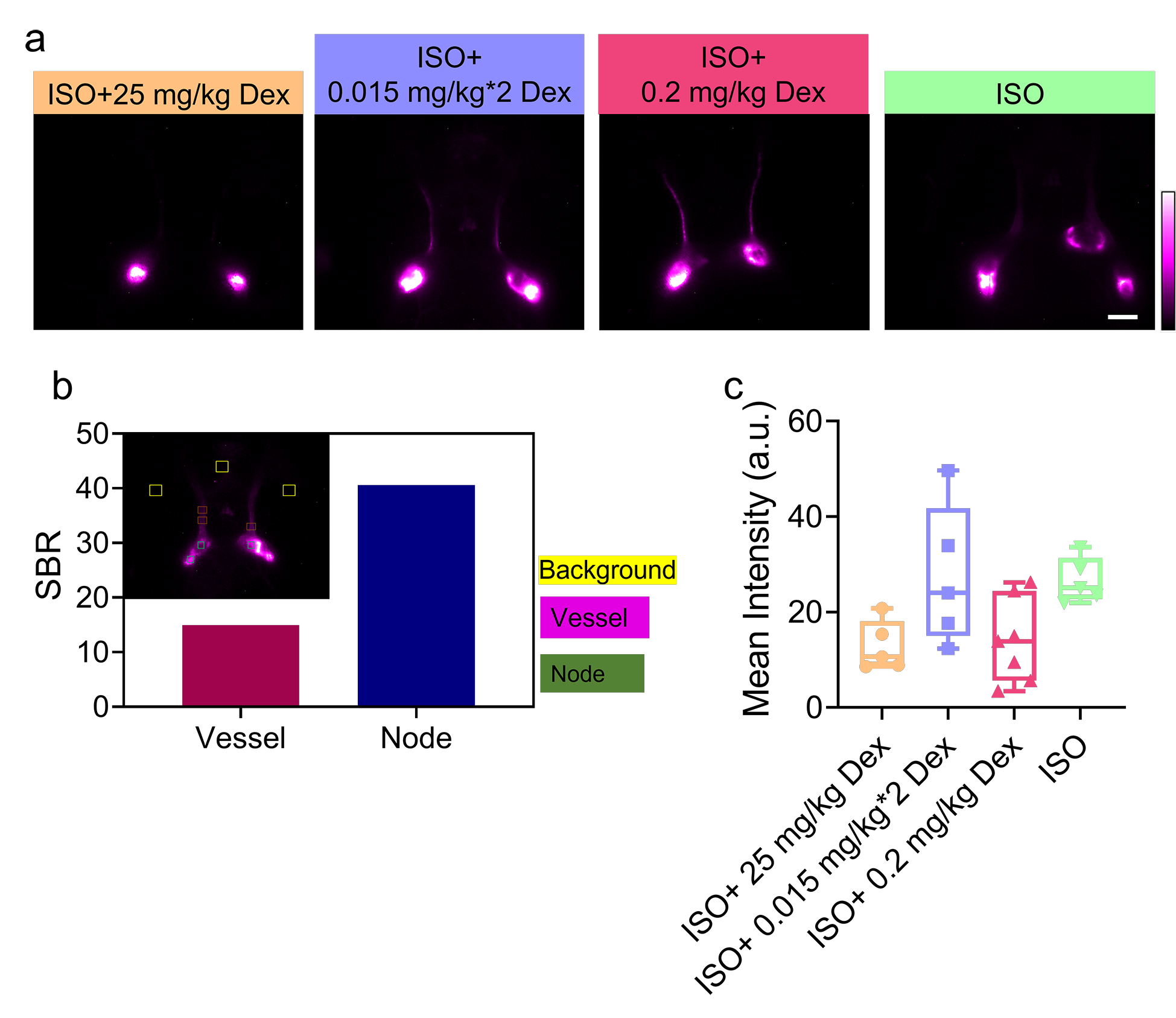


Figure S10. NIR-II images acquired through intact skin demonstrate the impact of anesthetic regimens on the influx of CSF in CNS-draining lymph nodes. a) Illustrative images of lymph nodes under various anesthetic regimens following the initiation of CM injection. b) Enhanced imaging of lymph vessels and lymph nodes with high contrast. c) Mean intensity of lymph nodes in all groups of anesthetized mice. n=5 for ISO plus Dex 25 mg/kg group, n=5 for ISO plus Dex 0.015 mg/kg*2 group, n=7 for ISO plus Dex 0.2 mg/kg group, n=5 for ISO group. White scale bar: 2 mm.


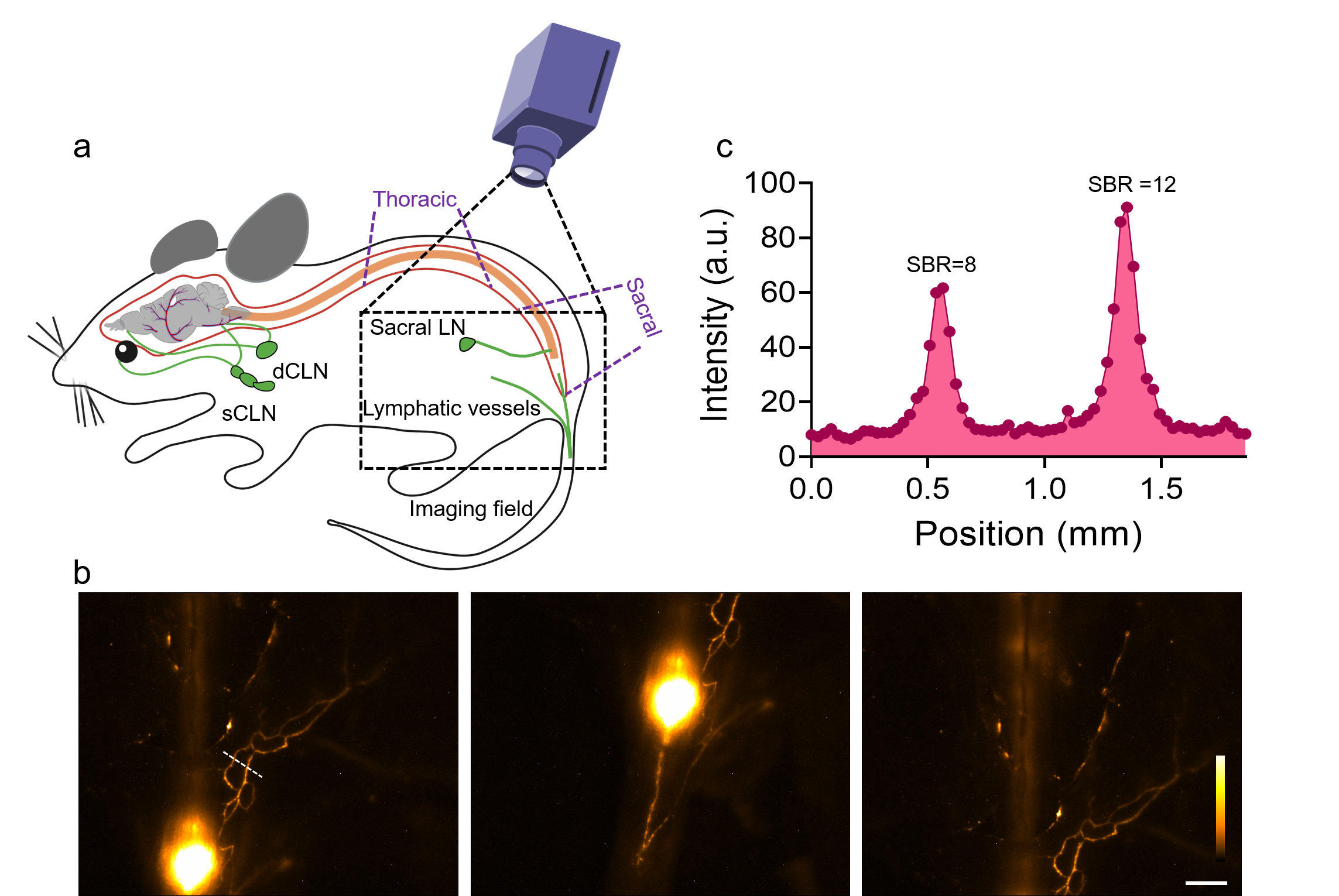


Figure S11. QDs as CSF tracers enable high-contrast imaging of the clearance of CSF through lymphatic vessels and LNs in the spine. a) Schematic of the imaging of the sacral spine clearance through lymphatic vessels. b) NIR-II imaging of different positions of the spine, spine-adjacent lymph nodes, and the connected lymphatic vessels. c) Cross-sectional profile of the lymphatic vessel plotted from the white dashed in b. White scale bar: 2 mm.


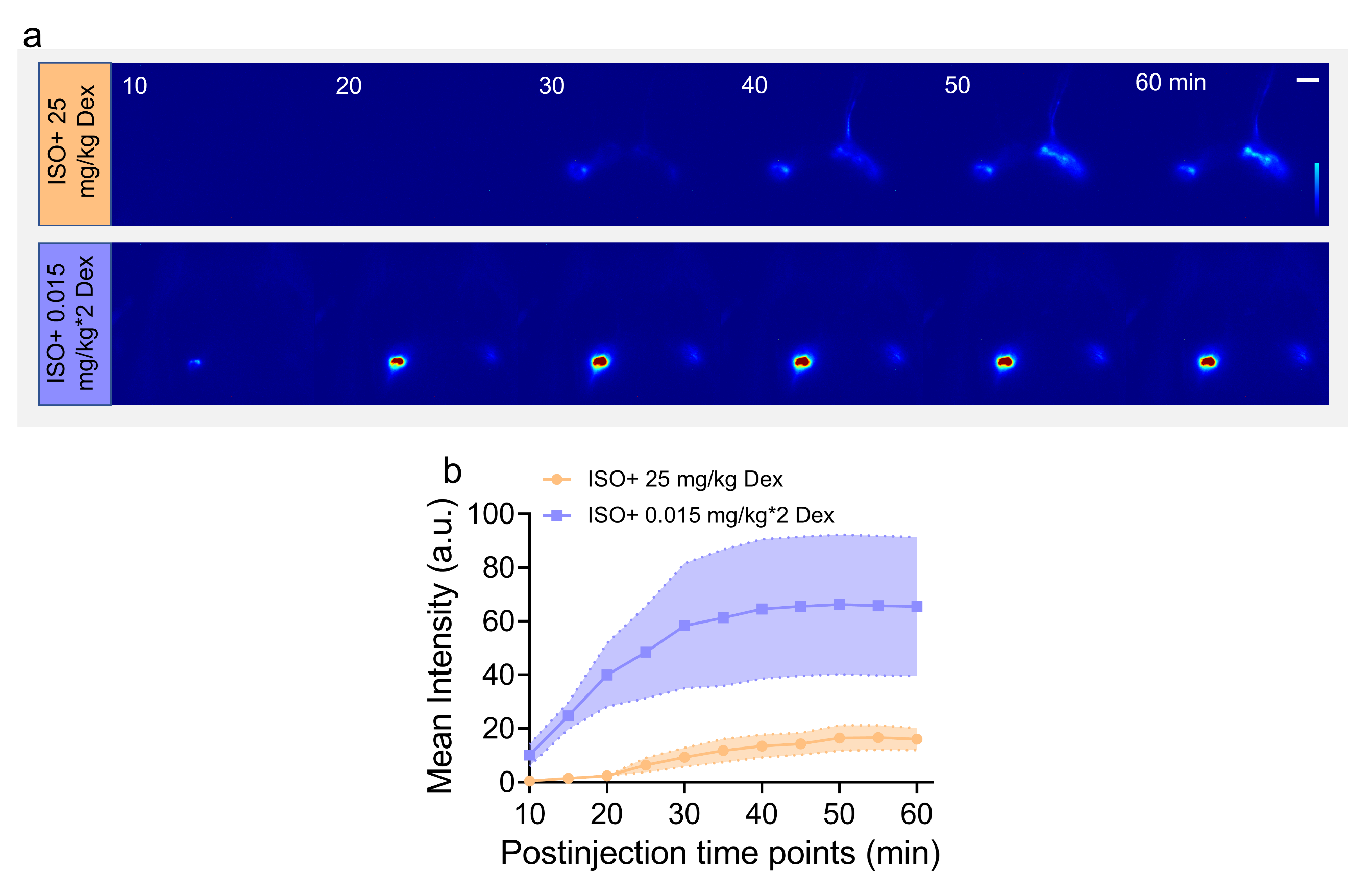


Figure S12. In vivo NIR-II imaging of CSF efflux into mandibular lymph nodes under the doses of Dex 25 mg/kg and 0.015 mg/kg*2, respectively. a) Representative images of NIR-II tracer-filled lymph nodes over 60 minutes. b) Quantification of fluorescence signal variation in lymph nodes over 60 minutes. n=5 for ISO plus Dex 25 mg/kg group, n=6 for ISO plus Dex 0.015 mg/kg*2 group. White scale bar: 2 mm.


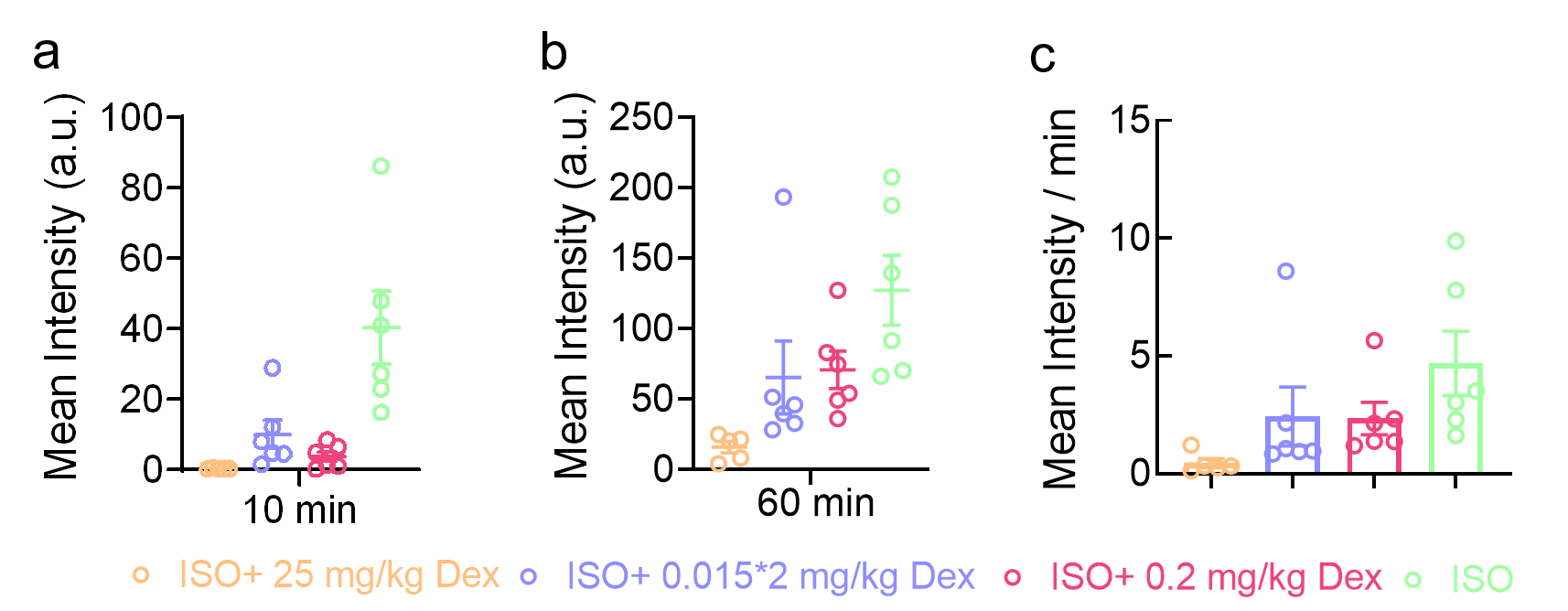


Figure S13. Comparison of lymph node intensity at a) 10 minutes and b) 60 minutes after the start of CM injection. c) Comparison of lymph node filling rate in all cohorts of mice. n=5 for ISO plus Dex 25 mg/kg group, n=6 for ISO plus Dex 0.015 mg/kg*2 group, n=6 for ISO plus Dex 0.2 mg/kg group, n=6 for ISO group. To facilitate a direct comparison, the data points for the ISO alone group and the ISO plus Dex 0.2 mg/kg group are displayed again from Figure 3c, d.


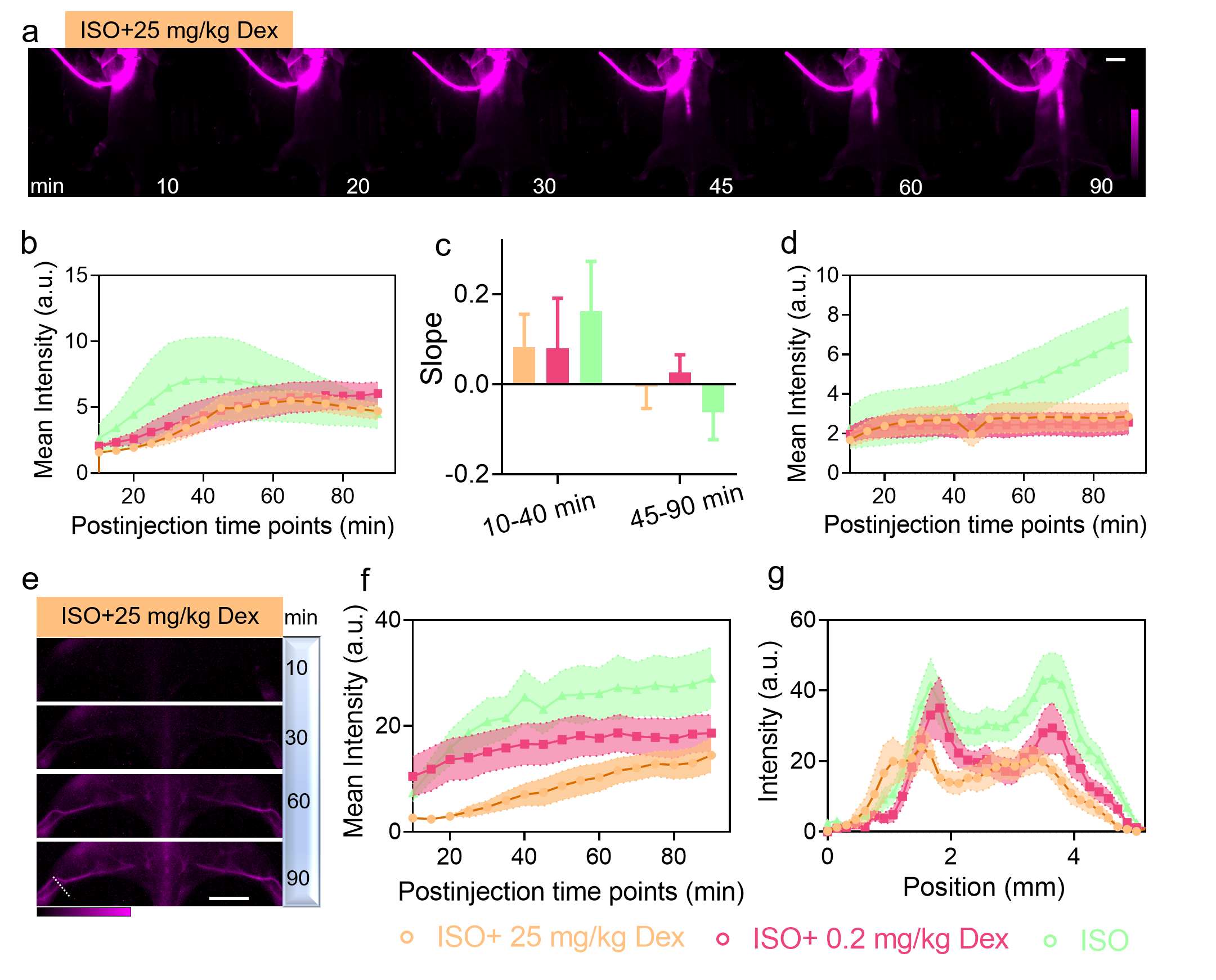


Figure S14. In vivo NIR-II imaging of CSF efflux into spines under different anesthesia regimens. a) Representative images showing the distribution of NIR-II tracer in the spine over 90 minutes using the ISO supplement with 25 mg/kg Dex anesthesia regimen. b) Quantification of fluorescence signal variation in the thoracic region over 90 minutes using three anesthesia regimens, n=6 for ISO plus Dex 25 mg/kg group, n=8 for ISO plus Dex 0.2 mg/kg group, n=6 for ISO group. c) Comparison of the rate of thoracic region filling before and after 40 minutes using three anesthesia regimens, n=6 for ISO plus Dex 25 mg/kg group, n=8 for ISO plus Dex 0.2 mg/kg group, n=6 for ISO group. d) Quantification of fluorescence signal variation in the sacral region over 90 minutes using three anesthesia regimens, n=6 per group. e) Representative images of hindlimb vessels using the ISO supplement with 25 mg/kg Dex anesthesia regimen. White scale bar: 1 cm. f) Quantification of fluorescence signal variation in hindlimb vessels over 90 minutes using three anesthesia regimens, n=5 for ISO plus Dex 25 mg/kg group, n=8 for ISO plus Dex 0.2 mg/kg group, n=6 for ISO group. g) Comparison of hindlimb vessel intensity at the 90-minute time point using three anesthesia regimens, n=5 for ISO plus Dex 25 mg/kg group, n=8 for ISO plus Dex 0.2 mg/kg group, n=6 for ISO group. To facilitate a direct comparison, the results for the ISO alone group and the ISO plus Dex 0.2 mg/kg group in b, c, d, f, g are displayed again from Figure 3g, h, i, k, l.


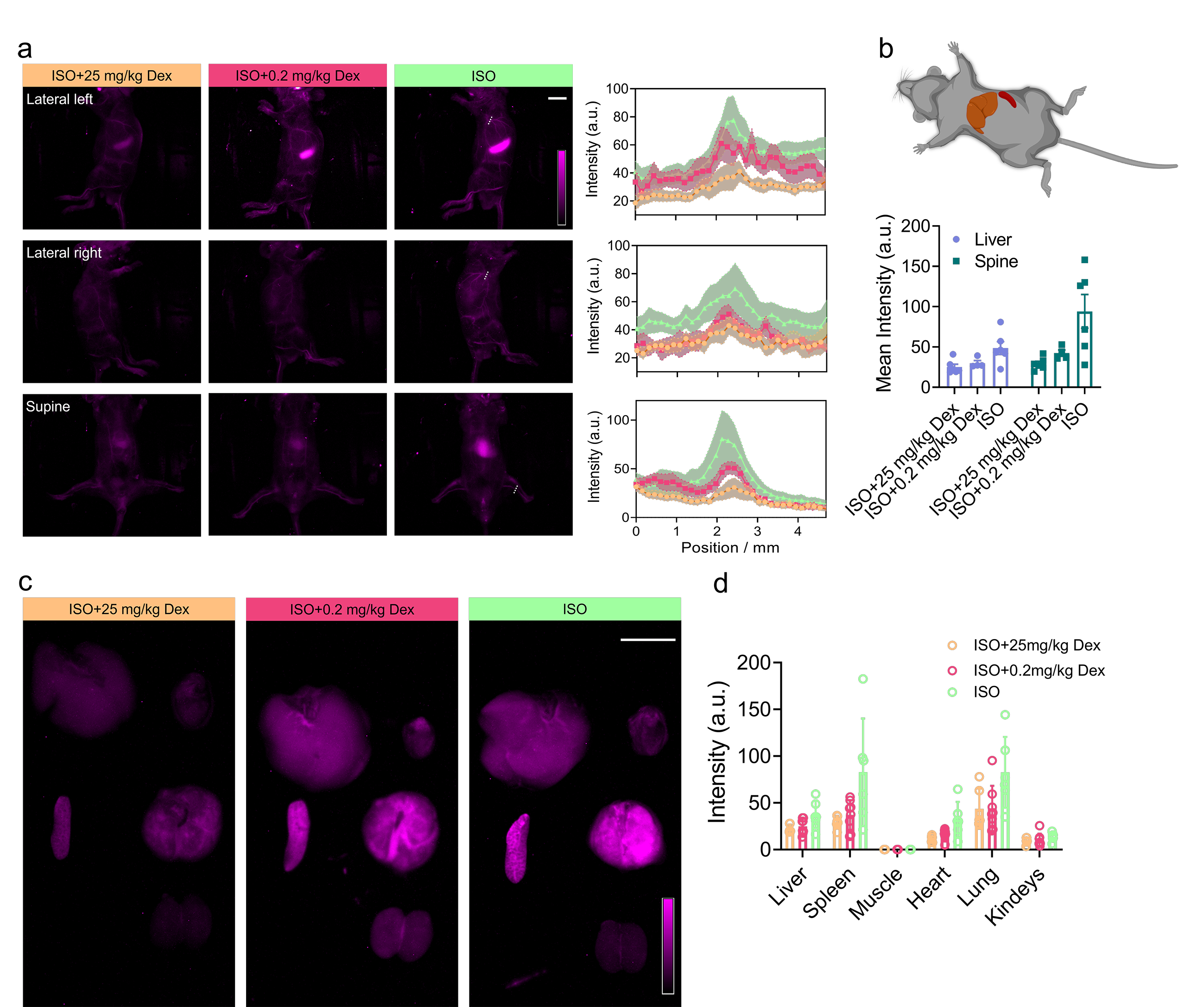


Figure S15. NIR-II wide-field imaging allows for the collection of intensity data on whole-body vessel networks and the NIR-II tracer distribution in major organs under different anesthesia regimens. a) Comparison of whole-body vessel network intensity at the 90-minute time point under three anesthesia regimens, lateral left: n=6 for ISO plus Dex 25 mg/kg group, n=4 for ISO plus Dex 0.2 mg/kg group, n=6 for ISO group, lateral right: n=5 for ISO plus Dex 25 mg/kg group, n=3 for ISO plus Dex 0.2 mg/kg group, n=5 for ISO group, supine: n=6 for ISO plus Dex 25 mg/kg group, n=4 for ISO plus Dex 0.2 mg/kg group, n=6 for ISO group. b) Comparison of mean liver and spleen intensity at the 90-minute time point under three anesthesia regimens, n=6 for ISO plus Dex 25 mg/kg group, n=4 for ISO plus Dex 0.2 mg/kg group, n=6 for ISO group. c) NIR-II imaging of main organs from different groups. White scale bar: 1 cm. d) Quantified signals from these three groups, n=6 for ISO plus Dex 25 mg/kg group, n=8 for ISO plus Dex 0.2 mg/kg group, n=6 for ISO group.

**
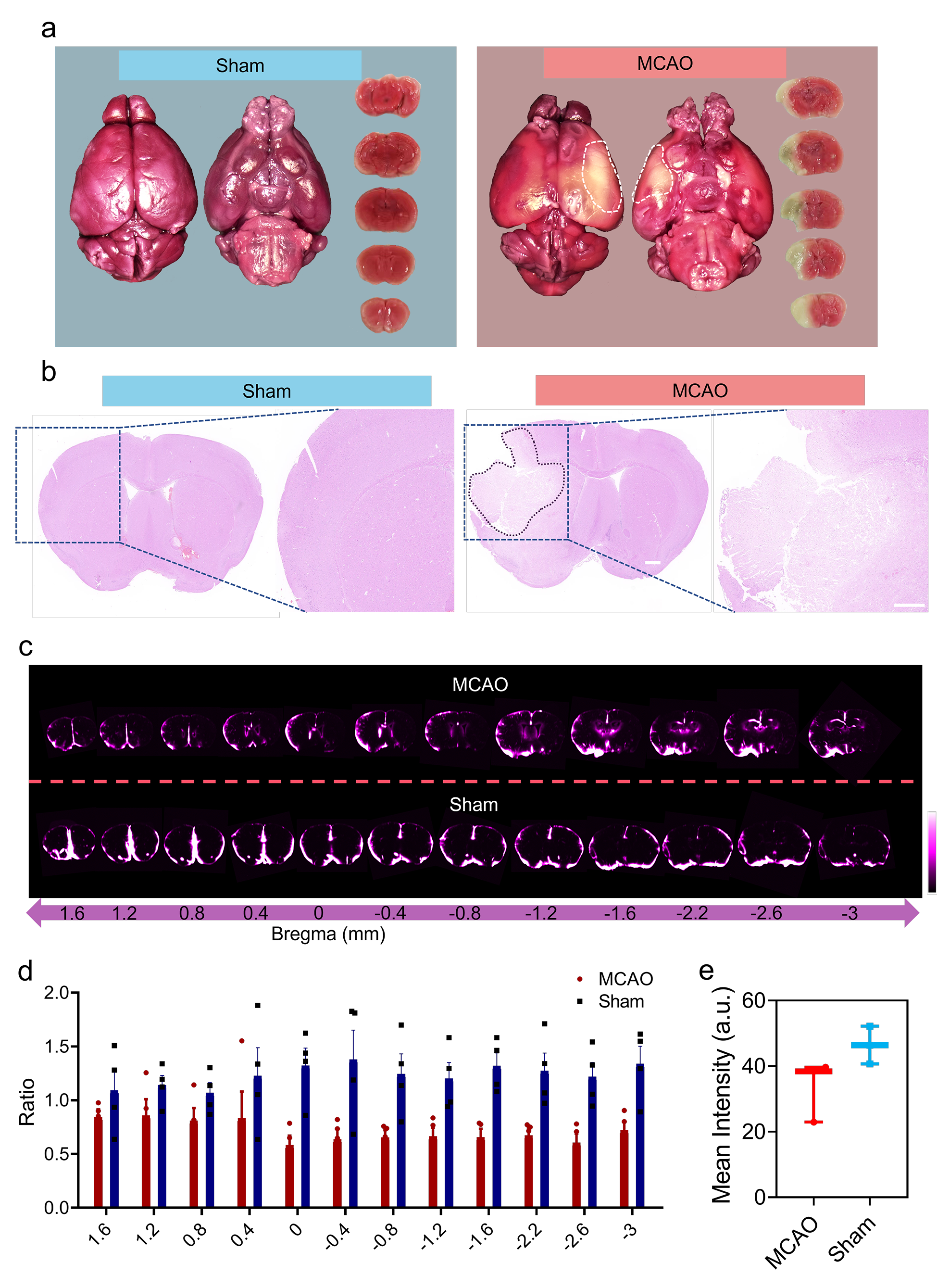
**

Figure S16. The suture-occluded method used to create the ischemia-reperfusion model. a) TTC stain of the sham group and MCAO group after 24 hours of reperfusion. b) The H&E stain of the sham group and MCAO group after 24 hours of reperfusion. White scale bar: 500 µm. c) Representative images of whole-brain slices for the sham group and MCAO group. d) The distribution of BSA@IR-780 in the anterior/posterior regions of the brain in the sham group and MCAO group, n=4 per group. e) Comparison of mean liver intensity between MCAO mice and sham mice 6 hours post-infusion, n=3 per group.

**References**

1. Zhang M, Yue J, Cui R, Ma Z, Dai H. Bright quantum dots emitting at 1,600 nm in the NIR-IIb window for deep tissue fluorescence imaging. Proc Natl Acad Sci. 2018;115:201806153.

2. Ma Z, Zhang M, Yue J, Alcazar C, Zhong Y, Doyle TC, Dai H, Huang NF. Near-Infrared IIb Fluorescence Imaging of Vascular Regeneration with Dynamic Tissue Perfusion Measurement and High Spatial Resolution. Adv Funct Mater. 2018;28:1803417.

3. Li H, Wang M, Huang B, Zhu SW, Zhou JJ, Chen DR, Cui R, Zhang MX, Sun ZJ. Theranostic near-infrared-IIb emitting nanoprobes for promoting immunogenic radiotherapy and abscopal effects against cancer metastasis. Nat Commun. 2021;12:7149.

4. Tian R, Zeng Q, Zhu SJ, Lau J, Chandra S, Ertsey R, Hettie KS, Teraphongphom T, Hu ZB, et al. Albumin-chaperoned cyanine dye yields superbright NIR-II fluorophore with enhanced pharmacokinetics. Sci Adv. 2019;5:eaaw0672.

5. Bai L, Hu Z, Han T, Wang Y, Xu J, Jiang G, Feng X, Sun B, Liu X, et al. Super-stable cyanine@albumin fluorophore for enhanced NIR-II bioimaging. Theranostics. 2022;12:4536-47.

6. Welsher K, Liu Z, Sherlock SP, Robinson JT, Chen Z, Daranciang D, Dai H. A route to brightly fluorescent carbon nanotubes for near-infrared imaging in mice. Nat Nanotechnol. 2009;4:773-80.

7. Zhu S, Herraiz S, Yue J, Zhang M, Wan H, Yang Q, Ma Z, Wang Y, He J, et al. 3D NIR-II Molecular Imaging Distinguishes Targeted Organs with High-Performance NIR-II Bioconjugates. Adv Mater. 2018;30:1705799.

8. Tian R, Ma H, Zhu S, Lau J, Ma R, Liu Y, Lin L, Chandra S, Wang S, et al. Multiplexed NIR-II Probes for Lymph Node-Invaded Cancer Detection and Imaging-Guided Surgery. Adv Mater. 2020;32:1907365.
